# Cysteine Oxidation in Human Galectin-1 Occurs Sequentially via a Folded Intermediate to a Fully Oxidized Unfolded Form

**DOI:** 10.3390/ijms25136956

**Published:** 2024-06-25

**Authors:** Hans Ippel, Michelle C. Miller, Ruud P. M. Dings, Anna-Kristin Ludwig, Hans-Joachim Gabius, Kevin H. Mayo

**Affiliations:** 1 Department of Biochemistry, Molecular Biology & Biophysics, University of Minnesota Health Sciences Center, 6-155 Jackson Hall, 321 Church Street, Minneapolis, MN 55455, USA; h.ippel@maastrichtuniversity.nl (H.I.); mill0935@umn.edu (M.C.M.);; 2 Department of Biochemistry, Cardiovascular Research Instutute Maastricht (CARIM), University of Maastricht, 6229 ER Maastricht, The Netherlands; 3 Department of Veterinary Sciences, Physiological Chemistry, Ludwig-Maximilians-University, 80539 Munich, Germany

**Keywords:** agglutination, alarmin, disulfide bridges, inflammation, lectin

## Abstract

Galectins are multifunctional effectors in cellular homeostasis and dysregulation. Oxidation of human galectin-1 (Gal-1) with its six sulfhydryls produces a disulfide-bridged oxidized form that lacks normal lectin activity yet gains new glycan-independent functionality. Nevertheless, the mechanistic details as to how Gal-1 oxidation occurs remain unclear. Here, we used ^15^N and ^13^C HSQC NMR spectroscopy to gain structural insight into the CuSO_4_–mediated path of Gal-1 oxidation and identified a minimum two-stage conversion process. During the first phase, disulfide bridges form slowly between C16-C88 and/or C42-C66 to produce a partially oxidized, conformationally flexible intermediate that retains the ability to bind lactose. Site-directed mutagenesis of C16 to S16 impedes the onset of this overall slow process. During the second phase, increased motional dynamics of the intermediate enable the relatively distant C2 and C130 residues to form the third and final disulfide bond, leading to an unfolded state and consequent dimer dissociation. This fully oxidized end state loses the ability to bind lactose, as shown by the hemagglutination assay. Consistent with this model, we observed that the Gal-1 C2S mutant maintains intermediate-state structural features with a free sulfhydryl group at C130. Incubation with dithiothreitol reduces all disulfide bonds and allows the lectin to revert to its native state. Thus, the sequential, non-random formation of three disulfide bridges in Gal-1 in an oxidative environment acts as a molecular switch for fundamental changes to its functionality. These data inspire detailed bioactivity analysis of the structurally defined oxidized intermediate in, e.g., acute and chronic inflammation.

## 1. Introduction

Interest in structural studies of lectins has considerably increased due to the realization of their abilities to ‘read’ glycan-encoded information of cellular glycoconjugates and ‘translate’ it into cellular activities [1]. In this field, the nature of modulatory events on activity and structure in terms of systematic profiling of ligand binding and protein folding has awaited clarification. An instructive example for the fundamental importance of such processes is provided by the alarmin high-mobility group box 1 protein (HMGB1) [2,3]. Intracellularly, its three cysteines are reduced allowing the protein to perform its nuclear function; upon non-classical secretion, HMGB1 associates with the chemokine CXCL12 to serve as a chemoattractant [4]. The formation of the C23-C45 disulfide bridge is the structural reason for HMGB1 to induce proinflammatory cytokines, explaining its designation as alarmin [5]. Adhesion/growth-regulatory ga(lactose-binding)lectins can also engage in multi-tasking (both intra- and extracellularly) following non-classical secretion and the ability to bind chemokines [6,7,8,9,10,11]. Intriguingly, this lectin group can undergo a redox-dependent change like HMGB1. Elucidating the nature of this change in human lectins is a prerequisite to defining biomedically relevant structure–activity relationships.

Several lines of evidence underscore the fundamental importance of cysteine residues in certain galectins: the need for a reducing agent such as dithiothreitol (DTT), the presence of a carboxamidomethylated derivative via treatment with iodoacetamide, and the strategic introduction of C-to-S mutations, which have been noted to maintain lectin activity. Most research has been conducted on mammalian galectin-1 (Gal-1) with its six cysteine residues [12,13,14,15,16]. As a cellular protein, Gal-1 can be reacted with glutathione [17] or hydrochloric acid and N-chloramines [18] to detect the presence of its thiol groups in cell extracts. The six Cys residues in human Gal-1 can be entirely substituted with Ser residues without loss of lectin activity [19,20], indicating the potential for regulatory processes rather than involvement in ligand binding. On the chemical level, the oxidation of thiol groups and the formation of three disulfide bridges yields a mass reduction of 6 Da for bovine Gal-1 [21], apparently converting reduced (intracellular) Gal-1 to the so-called oxidized Gal-1 that dissociates to its monomer state. This, in turn, leads to the loss of β-galactoside binding and acquisition of carbohydrate-independent growth regulation or axonal regeneration [19,22,23,24]. Obviously, a fundamental shift in quaternary structure and ligand binding properties occurs in a redox-dependent manner, as with alarmin (described above).

Structurally, the oxidation of Gal-1 causes a decrease in Trp fluorescence anisotropy [25], a change in far-UV circular dichroism indicative of a reduction in β-strand content [14,26,27], and a three-stage alteration in time-resolved Fourier transform infrared spectra [28]. Mass spectrometric (MS) analysis of peptic/tryptic peptides has provided evidence of intramolecular disulfide bond formation via C2-C130/C16-C88/C42-C60 for bovine and human Gal-1 [19,21] and C2-C16/C42-C60 for rat Gal-1 [29]. Chromatographically (via gel filtration), oxidized Gal-1 “adopts a number of different states” [30] and shows structural heterogeneity. Oxidized Gal-1 can also appear as a covalently linked dimer (via an inter-molecular disulfide bond) under certain conditions [15,27]. Of conspicuous physiological relevance, the redox process appears to be reversible depending on environmental conditions [14,27,30]. Unfortunately, crystallographic information as to how oxidized Gal-1 looks is not available. In contrast, this is the case for chicken galectin (CG)-1B, which was produced via duplication of the orthologous gene for avian Gal-1 to establish the paralogue pair CG1A/-1B [31]. Oxidation of this galectin leads to intra-(C2-C7) or inter-molecular (C7-C7′) disulfide bridges, with resulting dimeric proteins being distinguished by their shape and type of association [32]. In this case, establishment of this covalent bonding is favored by the local vicinity between cysteines. Given the prominent role of Gal-1 in acute and chronic inflammation, neurodegenerative diseases, and malignancy [33,34,35,36], this apparent gap in our structural knowledge on “oxidized Gal-1” has prompted us to follow the previous suggestion that “NMR studies in solution can help to clarify this problem” [37]. By applying a strategic combination of methods, most importantly NMR spectroscopy, our present data shed light on the molecular details of the conversion of Gal-1 to its oxidized form(s).

**Figure 1:**
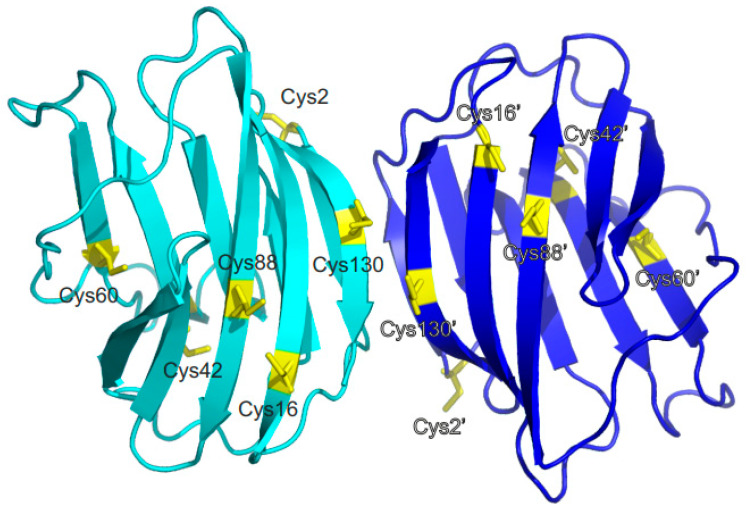
The positions of cysteine residues (C2, C16, C42, C60, C88, and C130) are highlighted on the X-ray structure of the Gal-1 homodimer to profile their distances (pdb access code: 1gzw). The lectin structure is presented such that the back side of the β-sandwich (F-face) is in the forward position and the lactose-binding S-face is below it. Proposed Cys residue pairs are indicated by dashed red lines, along with distances between paired Cys side chains, on one subunit of the Gal-1 dimer.

Figure 1 shows the distance profile of the six Cβ-SH atoms in human Gal-1. In order to pair cysteines, considering the MS data discussed above [19,21,29], distances of 4.1 Å (for C16-C88), 10.3 Å (for C42-C60), and 20.2 Å (for C2-C130) between the paired moieties were measured in the Gal-1 crystal structure. A structural model must explain how cysteines more than 20 Å apart can connect. Internal motions and conformational fluctuations can somewhat reduce these distances. Surface accessibility decreases in the order of C2-C130 to C16-C88 and then to C42-C60, as reflected in reactivity to iodoacetamide, with C2 being most reactive, followed by C130, then C88/C16, and barely, if at all, C42/C88 cysteines [14,38]. When exposed to β-mercaptoethanol during crystallization, the SH groups of C88 and C130, as well as C16 (if not oxidized to sulfenate), are always found to be covalently modified [39]. Within the dimer of Gal-1, the Cβ-SH atoms of the two C130 residues are only 4.1 Å apart (Figure 1), presenting the possibility for covalently linked dimers [15,27]. Because the ^1^H,^13^C,^15^N backbone and side-chain assignments were available for reduced Gal-1 [40], we performed heteronuclear single quantum coherence (HSQC) NMR spectroscopic experiments to monitor Gal-1 structural changes during the course of CuSO_4_-mediated oxidation.

Based on experimental evidence, we report here that Gal-1 undergoes a relatively slow conversion of C16 and C88 thiols, as well as of C42 and C60, to disulfide bridges, followed lastly by C2/C130 pairing. The initial structural transitions occur via a significant oxidation-mediated increase in the lectin’s internal motional dynamics, thus promoting the proximity of more distant cysteines. Our model for this non-random order of oxidation steps is supported by spatial proximity between cysteines and by site-directed mutagenesis. Our results confirm concept-based predictions. Hemagglutination assays indicate that complete loss of Gal-1 ligand binding activity occurs in the fully oxidized state, whereas the partially oxidized intermediate functions in essentially the same as the reduced lectin. Gal-1 ligand binding activity can be regained via the addition of DTT to oxidized Gal-1, underscoring the potential for an underappreciated on/off mechanism of the extracellular functionality of Gal-1 that depends on the nature of the environment, e.g., changes in oxidative capacity during inflammation.

## 2. Results and Discussion

**Figure 2:**
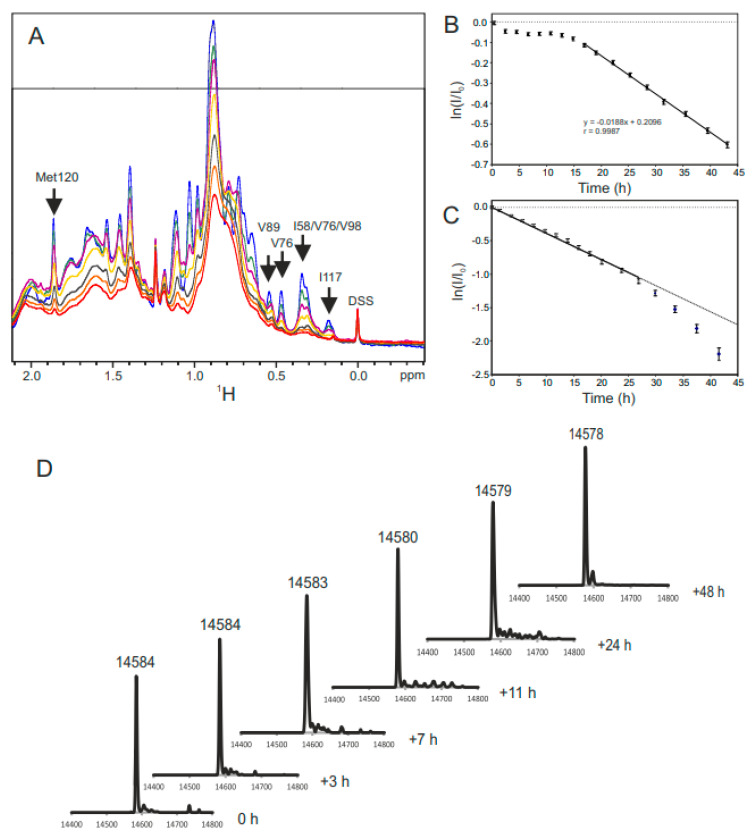
(**A**) A series of ^1^H NMR spectra for the methyl/methylene region of Gal-1 (20 μM) are shown at different time points during oxidation with 0.5 μM CuSO_4_, as given in detail. The upper right-hand corner insert presents the time-dependent change in overall ^1^H resonance intensity during the oxidation process. Colors used in these NMR spectral traces are for the following time points: blue (10 min), green (45 min), brown (90 min), yellow (2 h), black (3 h), orange (5 h), and red (10 h). Arrows indicate methyl peaks of assigned Gal1 amino acid residues. (**B**) Total integrated ^1^H signal in spectral region 2.52–0.07 ppm over time, showing a discrete change after ~15 h, ultimately leading to single exponential signal decay described by fit parameters shown in the plot. (**C**) Total integrated 1H signal in the narrower spectral region 0.41–0.08 ppm, containing only native (N) peaks of Gal-1, demonstrating nearly complete loss of natively folded dimer after 48 h oxidaton. (**D**) MALDI-TOF MS data are shown for Gal-1 (20 μM) as a function of oxidation time. The time points are given at the left side of each MS trace, and the change in mass can be followed using the numbers above each MS peak.

Gal-1 oxidation has been monitored via HSQC spectroscopy and MS. Disulfide bond formation in Gal-1 occurs extremely slowly (order of days) in the absence of reducing agents via air oxidation and much more quickly when catalyzed using CuSO_4_ as an oxidizing agent (order of hours) [17,24]. Figure 2A shows time-dependent changes in the methyl/methylene region of ^1^H NMR spectra of Gal-1 (20 μM) in the presence of 0.5 μM CuSO_4_ (30 °C). Several well-resolved methyl resonances were unambiguously assigned to I58, V76, V89, and I117, and their upfield positions indicate the proximity of their associated methyl groups to aromatic residues (ring current shift effects). Since these residues are located within the β-sandwich, chemical shifts in their resonances reflect the well-folded structure typical for this compact β-sandwich protein. As the incubation time under this oxidative condition proceeds, significant alterations arise in the NMR spectrum. These native-state, upfield-shifted methyl resonances, among others, are decreased in intensity, broadened, and/or shifted downfield, while new resonances appear and increase in intensity. The reduction in signal intensity is not the result of protein precipitation, because UV absorbance (220 nm) of the solution remains essentially constant at all stages, indicating that the protein stays in the solution with only its conformation being modified. Figure 2B plots the change in net integral of the methyl/ methylene 1H region resonating between 2.52 and 0.07 ppm vs. incubation time. During the first 15 h of incubation, the change is relatively small; after that, it occurs more rapidly through a single exponential decay. A similar plot but now integrating the narrower region 0.41 to 0.08 ppm, that only contains native Gal-1 methyl peaks (Figure 2C), show the distinct loss of folded structure reaching the 50% mark after ~12 h. Note that the resonances in these NMR spectra broaden over time, an effect that could result from the formation of molten globule states and/or reflect the presence of multiple oxidized states.

In order to figure out whether these time-dependent, NMR-based changes correlate directly with disulfide bond formation, corresponding evidence was obtained via MS for the occurrence of 2 Da losses due to (SH)_2_ to S-S conversions. These results are shown in Figure 2D. The MS trace of the native, fully reduced Gal-1 (0 h) shows a major *m*/*z* peak at 14,584 Da, as expected for the carbohydrate recognition domain (CRD) of Gal-1. As the period for oxidation increases, this *m*/*z* peak is decreased in value by three steps of 2 Da each; after 48 h, in the presence of 0.5 μM CuSO_4_ (or by 6 h with 4 μM CuSO_4_), the net reduction in the mass of Gal-1 is 6 amu (*m*/*z* = 14,578). This result is consistent with the generation of up to three S-S bonds from the six sulfhydryls in human Gal-1. In the absence of CuSO_4_, air oxidation can take up to about 1 week to cause the same effect, as seen in respective NMR spectra and MS runs.

The observation of an initially slow phase in Gal-1 oxidation followed by a faster phase change indicates inequality in the rate and position of formation of individual disulfide bonds. This suggests that disulfide bonds are formed sequentially and not randomly. Since internuclear distances between C_β_-SH atoms are conspicuously different in the range of 4.1 Å to 20.2 Å (Figure 1), there is a grading for oxidative reactivity consistent with MS data and chemical reactivity to iodoacetamide. In this regard, the C16-C88 pair is the best choice to initiate the process and, thus, to initiate the slow step in the cascade. Nevertheless, it is also plausible to form an inter-molecular disulfide bridge, as detected via gel electrophoresis of Gal-1 in the absence of β-mercaptoethanol [15,27]. Because this is especially true between C130 residues of subunits within the Gal-1 dimer (C130-C130’s distance is about 4.1 Å), we gave special attention to this point when analyzing our MS data. Thus, we incubated Gal-1 at a higher concentration of 30 μM under oxidative conditions and found no significant build-up of such a dimer. Even though we did observe a very small *m*/*z* peak at 29,168 Da, consistent with a covalently linked Gal-1 dimer, we must note that this is usually observed with native Gal-1 and remains constant in intensity during the entire time course of oxidation. However, for the production of oxidized Gal-1 in published axonal regeneration assays [19], different oxidation conditions were used (i.e., CuSO_4_ at 0.7 μM, Tris-HCl pH 8, 4 °C). If we oxidize Gal-1 (25 μM) under these conditions, the normally small Gal-1 dimer peak at *m*/*z* 29,166 is increased in intensity by 10–15%, indicating that covalent S-S Gal-1 dimers are likely formed.

**Figure 3:**
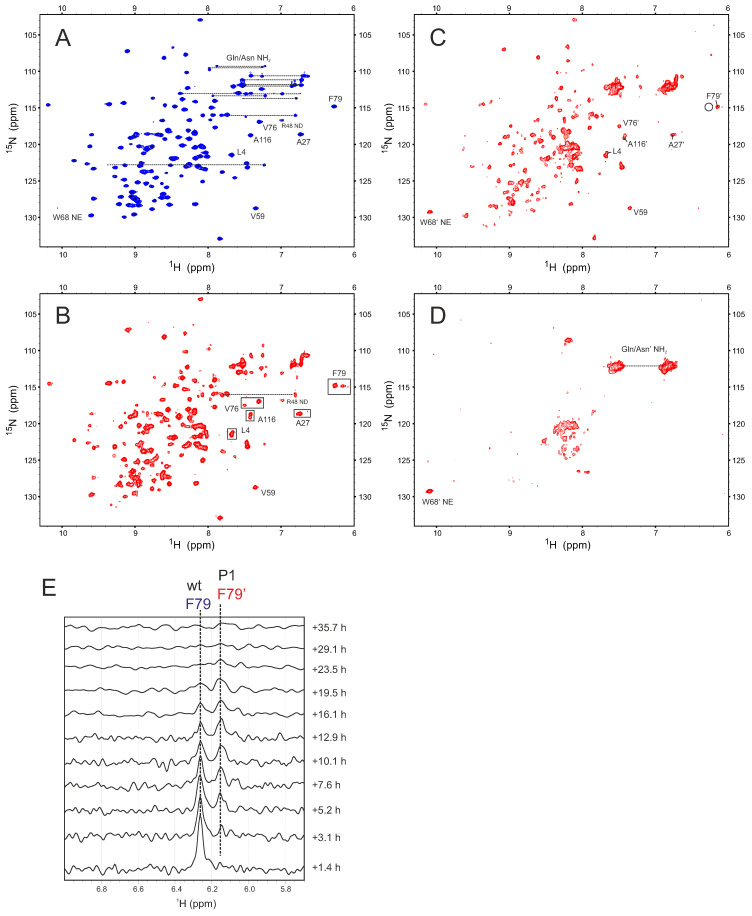
Four ^1^H–^15^N HSQC spectra of ^15^N-labeled Gal-1 are shown with selected cross-peak assignments. (**A**) Native Gal-1 (28 μM) prior to oxidation. (**B**–**D**) Gal-1 exposed to oxidative conditions for 3 h (**B**), for 12 h (**C**), and for 24 h (**D**). (**E**) Time dependence of spectral changes on F79 with slices through the ^1^H dimension of these HSQC spectra. ^15^N-labeled Gal-1 was dissolved in 25 mM potassium phosphate buffer (90% H_2_O/10% D_2_O), pH 7.3, 30 °C, and oxidative conditions (panels (**B**–**D**)) were established using 4 μM CuSO_4_.

Following further inspection of our HSQC data, we extended our insight into oxidation-induced structural changes. Figure 3 shows four ^1^H–^15^N HSQC spectra for the control (**A**) and three oxidation time points induced by 4 μM CuSO_4_ (i.e., 3 h (**B**), 12 h (**C**), and 24 h (**D**)). Compared to the HSQC spectrum of native Gal-1 (Figure 3A), oxidation brings about significant spectral changes, in particular, the appearance of new resonances in the initial slow phase of oxidation, during which intramolecular disulfide bond formation starts to occur, as shown by our MS results (Figure 2B). The appearance of multiple resonances is exemplified with F79 at 3 h incubation where two new resonances are observed (boxed in Figure 3B), the most upfield of which is relatively minor. As oxidation proceeds, the major new F79 resonance (F79′) grows in intensity as the native F79 resonance decreases in intensity. At the 12 h time point (Figure 3C), most HSQC resonances are broadened and only the new F79′ state is apparent, whereas the native state F79 resonance (black circle in Figure 3C) is not observed at this HSQC contour level. By 24 h (Figure 3D), when MS data indicate complete (or nearly complete) oxidation (i.e., all six cysteines become part of three disulfide bonds), most backbone resonances are too broad to be observed and those that arise from Asn and Gln side chains are still apparent. Extensive broadening of backbone NH resonances is likely explained by dynamic interconversion among the various Gal-1 conformers, a scenario indicative of a more open and/or unfolded protein structure than the native compact β-sandwich fold. What seems counterintuitive is that the presence of an intracellular disulfide bridge promotes backbone flexibility to Gal-1. Nevertheless, we have observed a similar phenomenon upon ligand binding to Gal-1 in which internal motions are enhanced upon lactose binding, thus increasing conformational entropy to lower the free energy of ligand binding [41]. As the oxidation process proceeds, intramolecular inter-cysteine distances above 10 Å or more were no longer an impediment to disulfide bond formation.

For a clearer view of F79 and F79′ resonance intensity changes, Figure 3E shows the time course of their spectral changes with slices through the ^1^H dimension of HSQC spectra. At the 3 h time point, the relative intensities of F79′ compared to native-state F79 are ~10% and ~90%, respectively. As oxidation proceeds, the intensity of F79′ increases, whereas that for the native state decreases. At about 12 h, the intensities of the F79 and F79′ peaks are nearly equal and a second new resonance, albeit minor, becomes apparent (see Figure 3B). The kinetics are consistent with what we observed in Figure 2A with a 50% change in net ^1^H resonance intensity occurring as well at ~12 h. At this 50% point, MS data indicate an average formation of two disulfide bonds (Figure 2B). These new resonances are most likely associated with Gal-1 in a partially oxidized state, where one or two intramolecular disulfide bonds reside in the CRD. If that were the case, then monitoring cysteine NH cross peaks should report valuable confirmatory information.

Supplemental Figure S1 shows expansions of ^1^H–^15^N HSQC spectra, where cysteine NH resonances for C16, C42, C60, C88, and C130 are observed. Indeed, these resolvable cysteine NH resonances are affected during the oxidation process, and this occurs in a rather similar manner even though the order of Cys pairing cannot be deduced unambiguously. NH cross peaks for C42, and possibly for C60, appear to be altered first, but this could simply be the consequence of resonance broadening due to changes in protein dynamics at those sites. Looking at chemical shifts, the C16/C88 pair appears in a prominent position, and resonances from C16 and C88 are the most shifted during oxidation. This suggests that a disulfide bond between these most spatially close residues is formed first during the initial oxidation phase. In that case, a C16S mutant should be rather insusceptible to oxidative stress (see below).

**Figure 4:**
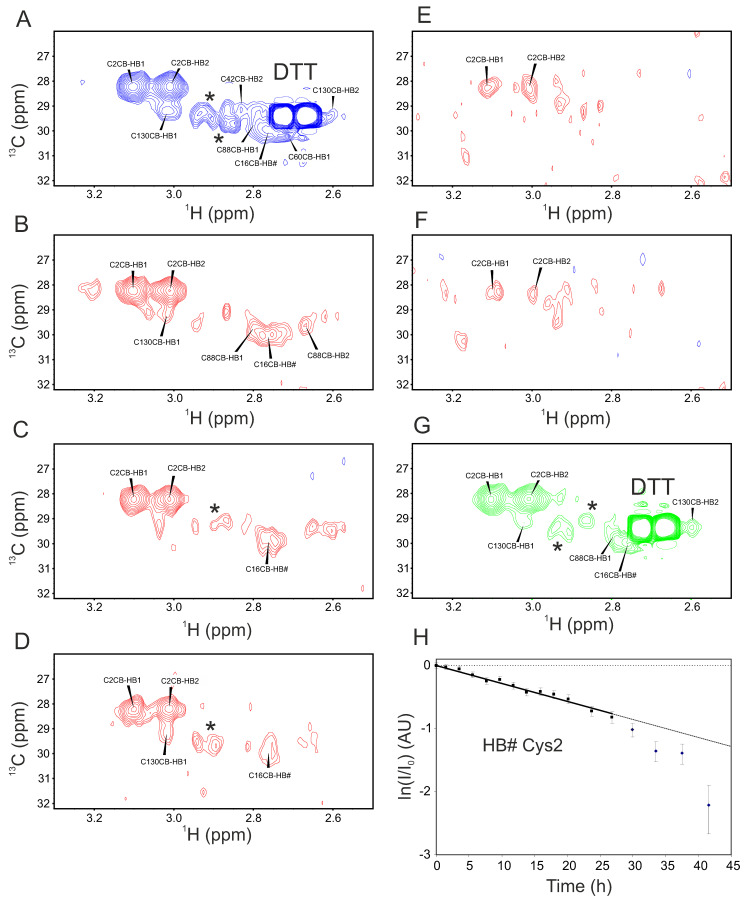
A series of ^13^C-based HSQC spectra for the cysteine methylene region of [^13^C,^15^N]-Gal-1 (28 μM) are shown at different time points during exposure to 0.5 μM CuSO_4_. The time points for the measurements are (**A**) zero as a reference (native Gal-1), (**B**) 1.5 h, (**C**) 5.6 h, (**D**) 14 h, (**E**) 18 h, (**F**) 34 h, and (**G**) 42 h. Following the removal of CuSO_4_ and 31 h long incubation in buffer containing 8 mM DTT and 2.5 μM EDTA, recovery to the native fold is 72%, based on normalized S/N from buffer containing ^1^H NMR spectra. The concentration of ^13^C,^15^N-labeled Gal-1 was 28 μM, dissolved in 25 mM potassium phosphate buffer (90% H_2_O/10% D_2_O), pH 7.3, and 0.5 μM CuSO_4_, and the solution was incubated at 30 °C. (**H**) Time-dependent changes in the integrated proton intensity of C2 methylene resonances are plotted, and the first part of the curve could be fitted to a single exponential decayrate as shown by the linear line. The asterix in panel A indicates unexplained peaks.

Whereas these data are not yet entirely predictive as to which disulfide bond is formed first, ^1^H–^13^C HSQC data indicate that C2 is the last cysteine to be oxidized. This important conclusion is based on the analysis of cysteine ^13^C_β_H_2_ resonances shown in Figure 4A–G. ^13^C_β_H_2_ resonances are observed for all cysteines, and most of these resonances are relatively broad, even in native Gal-1, most likely due to internal motions and/or conformational exchange. In this respect, the C_β_H_2_ resonances of C2 are exceptional, owing to their position at the relatively mobile N-terminus of Gal-1. The point to be made here is that no new C2 C_β_H_2_ resonances appear during the formation of the partially oxidized state(s). The only change measured is the decrease in their intensity during the oxidation process. Moreover, a plot of the fractional change in this intensity (plotted as ln [1-fraction intensity]) vs. time is linear over the first 28 h of oxidation (Figure 4H). This plot deviates from linearity at time points greater than ~30 h, something that could be dependent on other processes (see below). The apparent linearity over most of the oxidation process further supports our reasoning that the transition involving C2 is primarily a second-state process. In this case, the intermediate has a reduced C2 thiol group that finally becomes oxidized to generate the fully oxidized Gal-1. Overall, this data set strongly suggests that the C2-C130 bond is the final one of the three disulfide bridges to be formed. In that case, the C2S mutant of Gal-1 should maintain one free sulfhydryl at C130 (see below). Also, ^1^H–^13^C HSQC data provide further relevant information.

**Figure 5:**
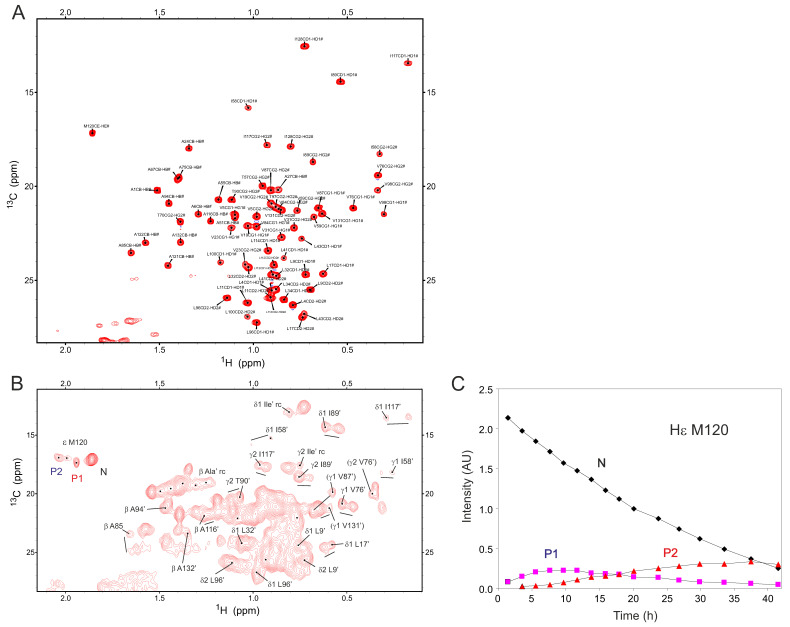
(**A**) Reference ^1^H–^13^C HSQC spectrum for assigned methyl peaks of natively folded dimeric Gal-1 in 8 mM DTT. (**B**) An expansion of the methyl region from a ^1^H–^13^C HSQC spectrum of Gal-1 is shown for the 18-h time point during oxidation, as discussed in the text. (**C**) The time-dependent change in intensities of the three major peaks observed for M120: “N” stands for native Gal-1, and P1 and P2 are oxidation states that have arisen over time following the removal of DTT. The data are consistent with a formation of the F79 intermediate state, shown in Figure 3, that builds up during exposure to CuSO_4_.

Supplemental Figure S2 shows expansions of the methyl region from these same ^1^H–^13^C HSQC spectra, acquired over the duration of oxidation. Resonances for I89, I117, M120, and I128 are boxed and labeled, and complete ^1^H–^13^C resonance assignments are provided in Supplemental Figure S3 for reference. As observed with our ^1^H–^15^N HSQC data, new resonances appear and increase in intensity during oxidation, whereas those for native Gal-1 decrease in intensity. Again, considering our MS data (Figure 2D), these new resonances must be attributed to various oxidized states of Gal-1, as intramolecular disulfide bonds are being formed. Figure 5A shows the ^1^H–^13^C HSQC methyl region for the 18 h time point, expanded with more resonances labeled. At this time point, four cross peaks for M120 are observed. The most upfield one at 1.87 ppm ^1^H is associated with the native state (labeled “N”), and the others (M120′) originate from different species of oxidized Gal-1 (labeled P1 and P2). The fourth M120′ peak is minor and not labeled. Other new resonances are associated with Ala, Thr, Ile, Leu, and Val residues (e.g., CH_3_ resonances from I58, I89, I117, L9, L17, L32, L96, V76, V131, V131, A94, A116, A132, and T90), representing sites throughout the protein.

Figure 5C plots the change in resonance intensities for these M120 signals vs. oxidation time. Although we can discern four peaks (or states) for M120 at 18 h oxidation, this number varies with the time or stage of oxidation. The most upfield resonance arises from native (N) Gal-1. Time-dependent intensity changes for N, P1, and P2 resonances are plotted in Figure 5B. However, because line widths for P1 and P2 are about 2-fold greater than those for N, the populations of P1 and P2 should be increased by about 2-fold relative to that of N. Thus, these data reveal that the population for the native state N decreases over time, as expected, whereas the populations for P1 and P2 first increase and then decrease in intensity during the oxidation process. P1 rises in intensity in the first oxidation phase, during which P2 displays an initial lag phase. These trends for P1 and P2 indicate a sequential (or serial) order of formation of Gal-1 oxidation intermediates, with the overall scheme being from N to P1, P2, and finally into O, the fully oxidized state. Although other intermediates aside from P1 and P2 are possible, they would be either very short-lived, highly broadened, and/or have very small populations below the level of HSQC detection limit.

For the N state, the decay curve is non-linear with respect to longer time points (Figure 2C) and requires the use of a double exponential function to be fitted optimally. This trend parallels that observed in ^1^H–^13^C HSQC spectra with cysteine C2 C_β_H_2_ resonances (Figure 4) and indicates that at least two events occur during oxidation of the N state, one event that is initially slow and the other(s) that subsequently proceed faster. This observation supports the sequential model introduced above. Although the initial part of the curve up to ~25 h can be fitted with a linear function, the remainder of the curve deviates substantially from linearity. Simple linear fits to initial and final parts of the curve show that the rates are only about a factor or two apart, i.e., ~0.7 × 10^−3^ min^−1^ and ~1.3 × 10^−3^ min^−1^, respectively. The initial slow rate of signal reduction for the N state is estimated to be the same as the initial rate of formation of P1, not indicating a lag in P2 formation. The conversion rate from P1 to P2 appears to be faster than that from N to P1. The final, fast oxidation phase starts at about 25 h after P1 has formed sufficiently and P2 production is underway. At this point, about two-thirds of N has already been converted, equivalent to forming of up to two disulfide bonds per Gal-1 monomer.

At the ~7 h time point, when the intensity of P1 is maximal and very little P2 has formed, our MS data (Figure 2D, which also shows slow and fast oxidation phases) indicate that the net Gal-1 mass has been reduced by only ~1–2 amu. Although there is, in principle, more than one way to explain the average, the easiest one is to consider 50% of Gal-1 molecules in the fully reduced state and 50% of them having only one disulfide bond formed. As mentioned above, our data indicate that Gal-1 is modified during the oxidation process (initial and later stages). At the initial stages of oxidation (slow kinetics phase), in which the native structure of Gal-1 is mostly preserved, albeit with increased dynamics, one possibility is that the dimer symmetry is broken by the formation of a single disulfide bond in one of the subunits. This is consistent with the appearance of P1, which reaches an equal (or near-equal) population with the N state and could indicate the formation of a Gal-1 heterodimer in which one subunit still is fully reduced and the other has one disulfide bond. An alternative explanation is that P1 represents an oxidized Gal-1 monomer species, i.e., the dimer is dissociated upon generating a single intramolecular disulfide bond. In that case, the resulting monomer would no longer function in the hemagglutinin assay (see below).

**Figure 6:**
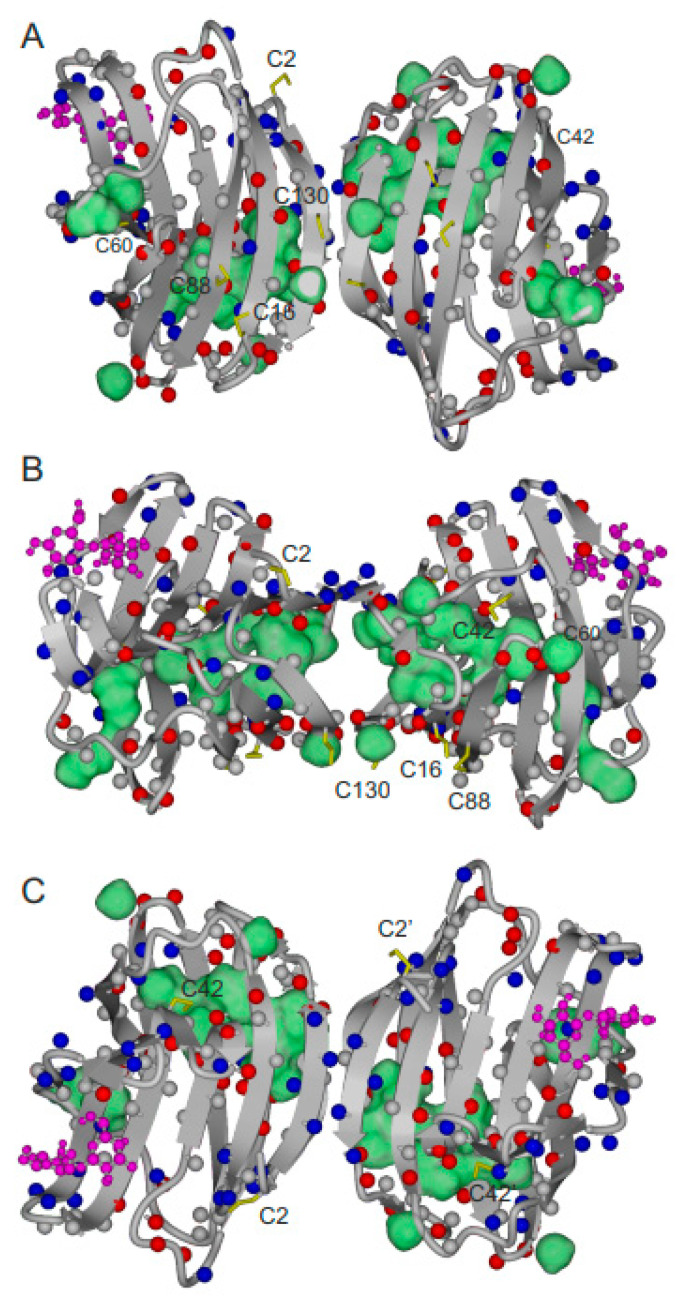
(**A**) Illustration of regions of resonance perturbation from exposure of Gal-1 (20 μM) to CuSO_4_: residues whose amide resonances are most perturbed (i.e., formation of double/multiple cross peaks and/or strongly reduced peak intensity) during the initial hours of oxidation are highlighted in red, and those that are only slightly perturbed are in blue. Grey dots indicate no change in peak position or relative intensity. In addition, the large green spheres indicate methyl groups whose resonances are most perturbed during the oxidation process. Cysteine residues are highlighted in yellow and labeled (orientation of the homodimer as in Figure 1). (**B**) Orientation of the Gal-1 homodimer shown in (**A**) is rotated by 90°. (**C**) Orientation of the Gal-1 homodimer shown in (**A**) is rotated by 180°, so that the β-galactoside-binding S-face is in front. Lactose molecules are shown in purple.

Regarding dynamics, our results serve to address the pertinent question as to the distribution of this parameter. In order to present the observed NMR spectroscopic data graphically, an overview of backbone NH groups that are most perturbed in ^1^H–^15^N HSQC spectra during the oxidation process is presented in Figure 6. Residues associated with resonances that are most perturbed (i.e., chemically shifted, formation of double/multiple cross peaks, and/or strongly reduced peak intensity) during the initial hours of oxidation are highlighted in red, and those that are only slightly perturbed are in blue. Grey dots indicate no change in peak position or relative intensity. Figure 6A has the F-face (the face of the β-sandwich opposite to the lactose-binding S-face) of the dimer in front. Figure 6B rotates the structure by 90°, and Figure 6C rotates the structure in Figure 6A by 180° to show a frontal view of the S-face. During the initial stage of the oxidation process, perturbations at backbone NH positions are not localized. Instead, they occur throughout the folded structure of Gal-1, as suggested by the ^13^C HSQC data on methyl groups (Figure 5). This suggests that the extent of internal motions within oxidized Gal-1 is generally increased, possibly promoting further oxidation by allowing more distant cysteines to come closer together.

An analysis of methyl resonances in ^1^H–^13^C HSQC spectra was added to further refine this picture. Most methyl groups of Ala, Thr, Ile, Leu, and Val side chains are located within the β-sheet sandwich. These include the M120 case discussed above, as well as, e.g., I58, I89, I117, L9, L17, L32, L96, V76, V131, V131, A94, A116, A132, and T90. Figure 6 highlights methyl groups (green spheres) whose resonances are most perturbed during oxidation. Even though some residues like I58, V76, and L96 near the lactose-binding site and relatively distant from any cysteine are perturbed at the initial stages of oxidation, the most perturbed methyls are close to C16 and C88, consistent with our Cys oxidation model. Many of these aliphatic side chains interact with each other through the β-sandwich interface. For example, side chains of V87 and I89, which flank C88 on β-strand 9, come into contact with those of L32 and L34 on the opposing β-strand 3. Also, the side chain of L17, flanking C16 on β-strand 2, interacts with that of M120 on the opposing β-strand 10. Moreover, intra-β-sandwich residues from all four strands contact each other. Since M120 is central to these interactions, its environment would be very sensitive (conformationally and/or dynamically) to the formation of the C16-C88 disulfide bond. Having detected only a few perturbations around C42 and C60 during the initial oxidation stage, it is most likely that the C16-C88 disulfide bond is formed first during Gal-1 oxidation, followed by C42-C60.

So far, we have shown that the formation of disulfide bonds in Gal-1 occurs in a sequential fashion, with the formation of the first one (probably C16-C88) having minimal, if any, effect on Gal-1 folding. Based on structural grounds, in order for the free sulfhydryls of the remaining cysteines C42-C60 and C2-C130 to come into proximity to facilitate Cys oxidation, there should, however, be a significant increase in conformational flexibility following the formation of the C16-C88 disulfide bond. Accordingly, resonances of intermediate oxidation states (e.g., see M120 P1 and P2 peaks in Figure 5) formed during the initial stages of Gal-1 oxidation exhibit broader line widths than in the native state, supporting the proposed increase in conformational exchange. Regrettably, our NMR data cannot inform on conformational changes that occur at later stages of oxidation, because the resonances are so highly broadened and, thus, unobservable. What is possible is to look at ligand binding during the early stages of oxidation.

**Figure 7:**
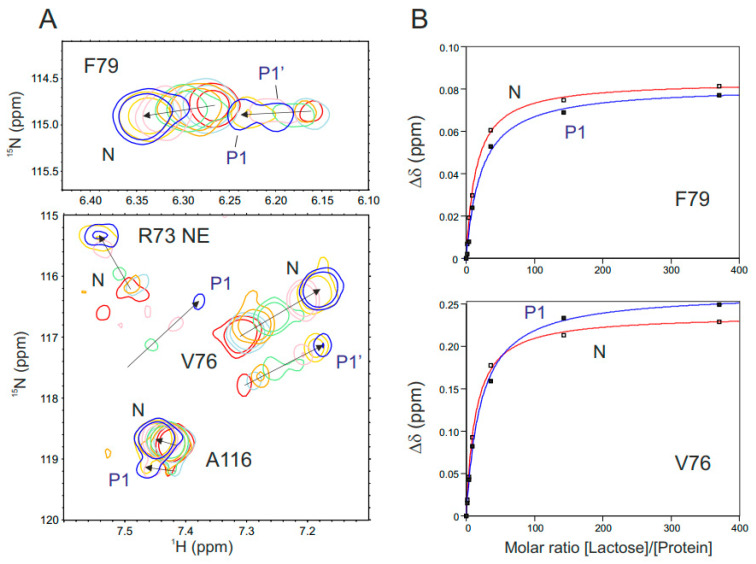
(**A**) Overlays of ^1^H–^15^N HSQC spectral expansions are shown for F79, R73, V76, and A116 for partially oxidized Gal-1 (12 h time point): one is for partially oxidized Gal-1 in the absence of ligand (black cross-peaks) and the others are for partially oxidized Gal-1 in the presence of lactose at 0.07 mM (green), 0.18 mM (magenta), 0.4 mM (blue), 2.0 mM (cyan), and 45 mM (red). Arrows indicate the direction of shifts upon increasing lactose concentration. Since Gal-1 is partially oxidized, some cross-peaks are doubled, representing native (N) and partially oxidized states P1 and P2 (or P1′). (**B**) Lactose titration curves are shown for ^1^H–^15^N-weighted chemical shifts for resonances of V76 and F79 (from native (N) Gal-1 (20 μM) and from partially oxidized (P1) Gal-1) vs. the concentration of lactose. The titration curves are fitted using a single exponential saturation binding function.

**Carbohydrate binding to partially oxidized Gal-1.** Because our HSQC data indicate that the native structure of Gal-1 is essentially preserved upon partial oxidation of Gal-1, we raised the question as to whether this state can still bind carbohydrates. To assess this, we performed a lactose titration with a sample of partially oxidized Gal-1, where resonances from both N and P1 states could be followed simultaneously. Overlays of ^1^H–^15^N HSQC spectral expansions are shown as a function of lactose concentration for F79, R73, V76, and A116 for the 12 h oxidation time point where N and P1 states are readily observed (Figure 7A). The contour in black is in the absence of lactose, and the contour in red is the endpoint at 45 mM lactose, with other colors for intermediate lactose concentrations, as stated in the legend of Figure 7. Because only F79 and V76 show clear changes during the lactose titration, Figure 7B plots ^1^H–^15^N-weighted chemical shift changes for these resonances vs. the [lactose]/[protein] concentration ratio. Based on these plots, it is apparent that the affinity for lactose is essentially the same for native (N) and the P1 partially oxidized state of Gal-1. We also found that the HSQC spectra of Gal-1 acquired in the presence or absence of 10 mM lactose look the same during the CuSO_4_-dependent oxidation process. The only difference is that the oxidation rate is reduced by about half in the presence of this saturating amount of lactose. Thus, the lactose-loaded state of Gal-1 attenuates the oxidation process.

**Figure 8:**
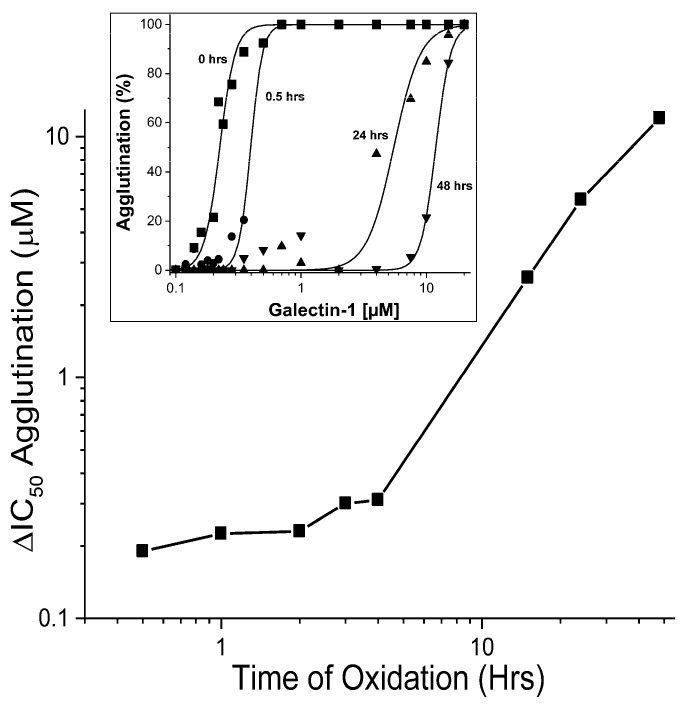
Agglutination of mouse erythrocytes was performed using a multi-well plate and at various concentrations of native Gal-1 and oxidized Gal-1, as exemplified in the insert. The extent of hemagglutination was quantified by reading the absorbance at 650 nm, A_650_, and data on the inhibition by lactose are obtained at eight time points.

Independently, the effect of oxidation on carbohydrate binding by Gal-1 was monitored using hemagglutination studies, which are traditionally used to provide insight into galectin-mediated cell–cell adhesion. Indeed, (ga)lectin-dependent bridging of glycoconjugates in cis/trans states underlies most aspects of lectin functionality [42,43]. Of note, this aspect of galectin activity will also report on the status of the Gal-1 dimer state, because our MS data (Figure 2D) exclude the generation of a covalently linked dimer under our oxidation conditions. Figure 8 plots IC_50_ values for agglutination vs. oxidation time, and the insert exemplifies the percent agglutination as a function of Gal-1 concentration, with data collected at three time points during the oxidation time course. Agglutination was quantified by plotting the absorbance at 650 nm (A_650_) as a function of Gal-1 concentration at each time point. Note that as the time of oxidation increases, the mid-point of each curve (IC_50_) shifts to higher Gal-1 concentrations. During the initial phase of oxidation, when mostly native and partially oxidized states of Gal-1 are present, IC_50_ values increase minimally. In contrast, IC_50_ values increase dramatically at later times as more Gal-1 is converted to the fully oxidized state. The time course for changes in agglutination IC_50_ values correlates well (regression coefficient R = 0.9) with that of the appearance of broad-resonance components in our NMR spectra (insert to Figure 2). The reduction in IC_50_ values during oxidation can be explained by increasing the amount of fully oxidized, and thus inactive, Gal-1, with partially oxidized Gal-1 remaining active. This observation is fully consistent with our NMR data in that partially oxidized Gal-1 maintains a native or native-like structure that can still bind lactose, whereas fully oxidized Gal-1 has lost its native structure and cannot bind lactose. In order to examine what happens to the quaternary structure, we added further NMR-spectroscopy-based data.

**Gal-1 dimers dissociate upon complete oxidation.** Our agglutination data presented in Figure 8 suggest that partially oxidized Gal-1 maintains bridging capacity. Otherwise, aggregation of red blood cells would not occur. At the quaternary structure level, the Gal-1 dimer state should, thus, be maintained. We confirmed this by performing pulsed field gradient (PFG) NMR diffusion experiments during oxidation, where mostly native and partially oxidized Gal-1 are present. The resulting diffusion coefficient (*D*) for native Gal-1 dimer (N) and for partially oxidized Gal-1 (P1) is essentially the same as ~1.05 × 10^−6^ cm^2^/s. For fully oxidized Gal-1, on the other hand, the *D* value is increased to 1.13 × 10^−6^ cm^2^/s. Although this *D* value is far from that of a Gal-1 monomer (*D* = 1.35 × 10^−6^ cm^2^/s), it does move in that direction. The adoption of an elongated shape, as suggested for Gal-1 based on dynamic light scattering [30], for CG-1B based on gel filtration and ultracentrifugation [32], and for dimeric CG-2 [44,45], may underlie this phenomenon. In any event, fully oxidized Gal-1 is not a well-folded CRD but rather a variant with its own hydrodynamic properties that can interact with new function-promoting partners but not with β-galactosides. Considering the physiological significance of fully oxidized Gal-1 (e.g., in late-stage inflammation where it can inhibit the proliferation of activated T lymphocytes and trigger interferon-γ-mediated apoptosis [46] thereby limiting the duration of the effector activity of this aspect of immune defense), the question arises as to whether the Gal-1 oxidation process is reversible or not.

**Native Gal-1 structure recovered from oxidized Gal-1.** The structure and activity of native Gal-1 can indeed be recovered from fully (or partially) oxidized Gal-1 via removal of the oxidizing agent CuSO_4_, and subsequent incubation with a reducing agent (i.e., DTT). Supplemental Figure S2 illustrates HSQC spectra acquired sequentially overnight, starting with native Gal-1 (S2**q**) in buffered solution with 0.5 mM CuSO_4_ and ending with fully oxidized Gal-1 ~41 h later (S2q**Q**). When the Cu_2_SO_4_ is neutralized with EDTA and 8 mM DTT is added to the solution, the sample of fully oxidized Gal-1 reverts to its native state. Basically, the HSQC spectrum of reduced oxidized-Gal-1 (S2**Rr**) looks no different from that of native Gal-1 (**A**). In addition, lactose binding and glycan-dependent cross-linking activity are also fully regained.

**Figure 9:**
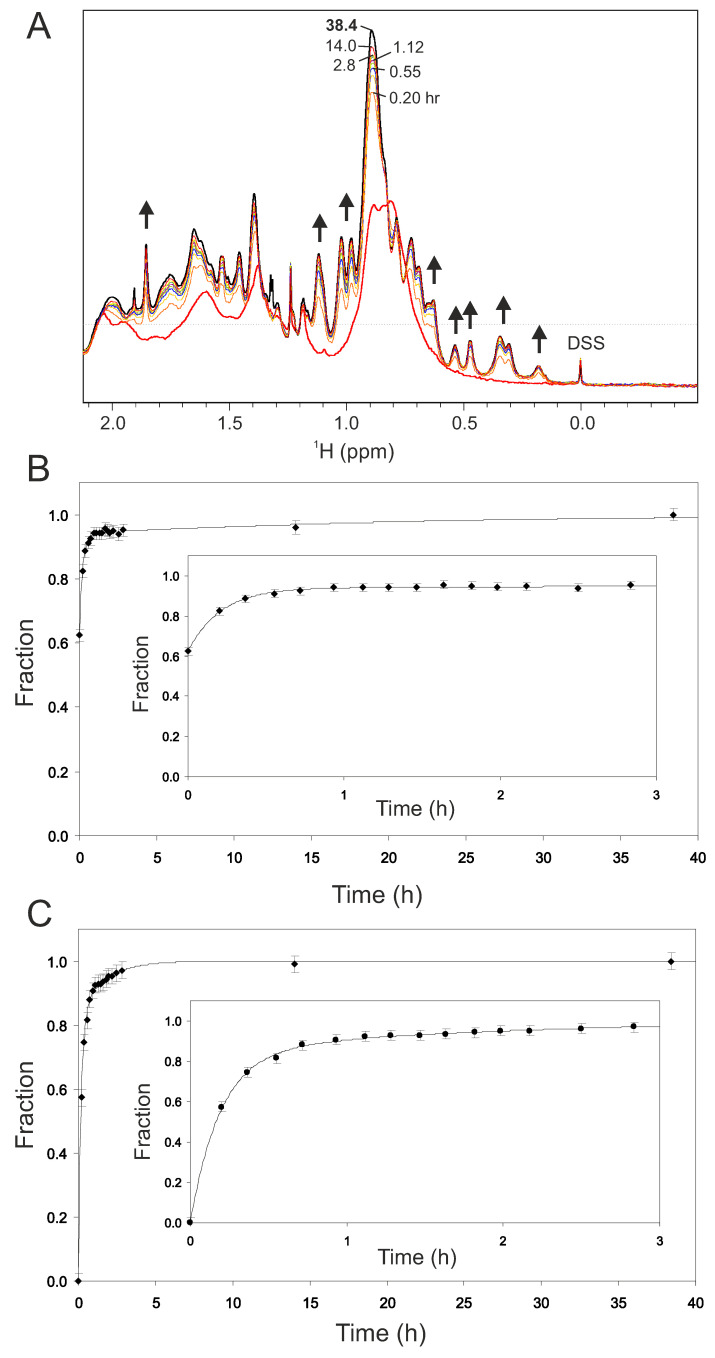
(**A**) 1D ^1^H NMR spectra of oxidized (4 μM CuSO_4_) Gal-1 after recovery to the native state in 8 mM DTT and 0.2 mM EDTA (30 οC). Thick red line indicates the starting NMR solution of fully oxidized Gal-1 spectrum (28 μM). Spectra after DTT recovery are displayed after 0.20, 0.37, 0.55, 0.72, 1.12, 1.98, 2.83, 14.0 and 38.4 h (thick black). After 0.20 h more than half of the oxidized species is already converted to its native structure. Arrows indicate the reoccurrence of natively folded (N) Gal-1. (**B**) Kinetic plot of total integrated 1H signal between 0.07–2.52 ppm as function of DTT incubation time, (**C**) Same type of plot, but here only for proton signals of defined natively folded (N) Gal-1 methyl peaks resonating between 0.08–0.41 ppm. Curves are fitted to a double exponential function having a fast and slow growth phase.

The recovery kinetics can be followed by analyzing ^1^H NMR spectra acquired over time starting with adding 8 mM DTT to the sample of oxidized Gal-1 (Figure 9A). Within the first 10–15 min upon the addition of DTT, most of the spectrum of oxidized Gal-1 has been converted to that of native or native-like Gal-1, with the remainder of the conversion taking considerably longer. An initial fast recovery phase is followed by a slower one, as the oxidation route is reversed. We quantified these kinetic phases by integrating resonance intensities during the time course of recovery. Two spectral regions were considered: case 1 with integration performed from 2.52 ppm to 0.07 ppm, where methyl groups and some methylene groups from all states (native, partially oxidized, and fully oxidized) resonate (Figure 9B); and in case with integration performed from 0.41 ppm to 0.08 ppm, where methyl resonances of I58, V76, V98, and I117 alone are found (Figure 9C). The methyl groups of I58, V76, V98, and I117 are located within the β-sandwich of native Gal-1 and, therefore, are suitable sensors for the well-folded structure of native Gal-1. For both cases, inserts are provided to show greater detail as to what happens over the first 3 h of recovery, during which >95% of native (or native-like) structure is recovered. These curves were fitted with single and double exponential functions. The formula of the double exponential function is shown in Equation (1):

y = 1 − [p_1_e^−k1t^ + p_2_e^−k2t^] (1)

where p and k stand for population and rate constant, respectively. Fits to these data with the double exponential function are shown in Figure 9B,C. While a single exponential fit to the data for case 2 was acceptable, with a regression coefficient of R = 0.98, the fit was less than optimal for case 1, with R = 0.87. Nevertheless, the double exponential fit gave satisfying data in both cases, with R = 0.998 (Table 1). 

This analysis indicates that 83% of the native (or native-like) structure is rapidly recovered in both cases. The partitioning of 83% (fast recovery) and 17% (slow recovery) may be explained by once again considering the total of six disulfide bonds in the native Gal-1 dimers and reversing the order in the sequential model discussed above. In this regard, the rapid reduction of five disulfide bonds per dimer would account for the population of 83%. Our data show that, within the rapid recovery phase, certain disulfide bonds are very quickly reduced, promoting the formation of native-like dimers. One disulfide bond (presumably one of the two C16-C88 bonds in the dimer) remains longer intact and is more slowly reduced. The 12-fold difference in the slow recovery rate for native-state methyl groups I58, V76, V89, and I117 compared to other methyl and some methylene groups could be attributed to the presence of both rapid and slow conformational re-shuffling, respectively, as the native-state structure returns. It is possible that I58, V76, V89, and I117 are involved in building the native folding core more rapidly than other aliphatic residues. Recovery of the native structure from oxidized Gal-1 is supported by MS data in which we observe a corresponding mass increase following DTT treatment of oxidized Gal-1. With rat Gal-1, the shift “in the trough in the circular dichroism spectrum going from 216 nm for native lectin to 207 nm for oxidized lectin” was indicative of substantial secondary structure alteration; “the shift was reversed by reduction with DTT (unpublished results)” [14]. However, reversibility can depend on the “circumstances” [26] and, thus, may only be partial [47] or involve protein aggregation that limits the extent of recovery [27], or even fail to occur for certain species [30].

**Testing our sequential oxidation model using mutagenesis.** Based on our data, we propose that oxidation is a stepwise, non-random process. The C16-C88 disulfide bridge likely forms first, causing conformational alterations that bring C42 and C60 into proximity, allowing the formation of the second disulfide bond. This partially oxidized state promotes further conformational shifting and dynamic changes to bring C2 into proximity with C130 to form the third S-S bond. At this stage, oxidized Gal-1 becomes “unfolded”. This process is reversible upon reduction of the oxidized state. To test this hypothesis, we produced two Gal-1 Cys-to-Ser mutants, i.e., C2S and C16S. The C2S mutant would be unable to form the final disulfide bridge, yet it should pass through the first phase of oxidation before becoming arrested prior to the final oxidation step. In contrast, the C16S mutant should be insensitive to oxidation if the C16 sulfhydryl indeed ignites the oxidation pathway. As with native Gal-1, ^1^H–^15^N HSQC data with each of these ^15^N-enriched mutants were determined as a function of oxidation time.

With the C2S mutant, HSQC spectra changed, as seen before in the initial phase of Gal-1 oxidation. However, as predicted, the broadened component characteristic of phase 2 was not observed, and the NMR signal was not degraded over time. The final “unfolded” state need for native Gal-1 to be attained could not be reached using the C2S mutant. As a sensor for detecting the remaining free sulfhydryls, application of Ellman’s reagent indicated that, as expected, one free SH group (i.e., C130) was present at the end of the oxidation process. The marked stability of the C2S mutant, in terms of binding activity seen by Hirabayashi and Kasai [3], is fully in line with the possibility of phase 1 transitions that do not impair lectin activity. Due to the stability of the C2S mutant, we could readily follow intensity changes for F79 N and P1 states, without concern for further oxidation of the P1 state. Supplemental Figure S4A shows the oxidation time course for N and P1 states from 2 to 40 h. During the oxidation process, the N state decreases in intensity, while the P1 state increases in intensity until it is the only observable state remaining. 

Because the Gal-1 C2S and C16S mutants appeared to be relatively stable in solution, we tried to elucidate their P1 3D structures by using NMR. We first made a C2S sample and started running an ^15^N-edited NOESY experiment. However, the following morning, we observed that the signal had degraded significantly, and upon looking at the NMR tube itself, it was apparent that the sample had mostly precipitated overnight, making structural elucidation impossible. However, the broadened, aggregated spectrum can be rescued partially by increasing the temperature from 30 to 40 °C (Figure S6). Because of this, we ran a CD spectrum of C2S and observed that its CD trace was essentially the same as that of native, reduced Gal-1 (Nesmelova et al., 2010). Therefore, it appears that C2S maintains the secondary structural characteristics of Gal-1, with its structure being more dynamic and possibly like that of a molten globule state. This is consistent with our hemagglutination data (Figure 9), which show that at least some activity remains up to the formation of this partially oxidized state. The same can be said for the C16S mutant.

The C16S mutant displays even more delayed kinetics of its P1 state (Figure S4B) even at 4 μM CuSO_4_, having a short ca. 10 h time window where the metastable oxidized state becomes conformationally pure to be structurally studied by NMR (Figure S5).

This evidence argues strongly in favor of C16-C88 bond oxidation, initiating structural/dynamical conversions. Both of these cysteines had been previously identified as critical via mutational analysis [27]. Because no oxidation-dependent conformational alteration then appears to occur, the C16S mutant could substitute for a chemically stabilized wild-type Gal-1 in functional assays, unlike the C2S mutant, which can still be converted to an intermediate. However, the C-to-S substitution at position 2 changes the orientation of the side chain of D123 by 180° and also the thermodynamics of lactose binding [39]. The role of C16 as a molecular switch is underscored by its high level of conservation in mammalian galectins, and cases like CG-1A (without C2 and C130, but still susceptible to oxidative damage to lectin activity) give incentive to further studying the role of individual constellations of cysteines in galectin activity in vertebrates.

## 3. Materials and Methods

### 3.1. Gal-1 Preparation and Isotopic Labeling

Uniformly ^13^C- and ^15^N-labeled Gal-1 was produced in BL21(DE3)-competent cells (Novagen), that were grown in minimal media and supplemented with either [^15^N]NH_4_Cl or [^13^C]glucose. Protein pellet was purified via affinity chromatography on lactosylated Sepharose 4B, and further fractionated on a gel filtration column, as previously described [40,41]. Normally, about 44 mg pure protein was obtained from 1 L of cell culture. Mutagenesis was carried out to obtain the C2S/C16S mutants, as described [39]. The purity of the final product was determined via one- and two-dimensional gel electrophoresis and MS analyses. Functional activity was assessed by using a T-cell death assay.

### 3.2. NMR Spectroscopy

NMR experiments were carried out at 30 °C on Bruker NMR spectrometers operating at ^1^H frequencies of 700, 850, or 900 MHz and equipped with H/C/N triple-resonance cold probes and x/y/z triple-axis pulse field gradient units. Gal-1 was dissolved at a concentration of ~25 μM in 20 mM potassium phosphate buffer at pH 7.0 in a 95% H_2_O/5% D_2_O mixture. Uniformly ^15^N-enriched or ^15^N/^13^C-enriched Gal-1 are used to perform HSQC NMR experiments. Raw data were converted and processed using NMRPipe [48] and analyzed using NMRview [49]. Lactose binding affinity was determined using ^1^H-^15^N HSQC spectra on ^15^N-labeled Gal-1, as a function of ligand concentration.

Diffusion coefficients were derived from pulsed field gradient (PFG) NMR experiments acquired on a Bruker Avance III 700 MHz spectrometer equipped with an H/C/N triple-resonance probe and x/y/z triple-axis pulse field gradient unit. The maximum magnitude of the gradient, g, was calibrated using deuterated water as the standard. Measurements were performed as previously described [50,51]. The value of the diffusion coefficient was estimated from the diffusion attenuation of the spin echo by using Equation (2).

A(g^2^) = A(0) exp (−γ^2^ δ^2^ g^2^ D t_d_), (2)

where γ is the gyromagnetic ratio for protons; δ is the duration of the pulsed field gradient, and t_d_ is the diffusion time, which comprises all time delays between pulses, during which the magnetization is aligned along the *z*-axis.

### 3.3. Mass Spectrometry

MALDI-TOF MS data were acquired for Gal-1 samples (native and oxidized). The protein was first desalted using C-4 Zip Tips (Millipore). For the matrix, A 3-(4-hydroxy-3,5-dimethoxyphenyl)prop-2-enoic acid was used and mixed with the Gal-1 samples (1:1 *v*/*v*) in 50% acetonitrile with 0.1% TFA. The Gal-1-matrix solution (1 L) was spotted on stainless-steel MALDI target plates and dried at room temperature. Analysis of the protein samples was performed on a Biflex III, Bruker Daltonics MALDI-TOF instrument (*m*/*z* range 5000–22,000 in reflection mode using a pulse frequency of 4.993 kHz. Ionization was induced by using a 337 nm N_2_ gas laser source at 8 lJ and 20 Hz repetition rate. Each MS trace was an average of 200 to 300 laser shots. The MS instrument was calibrated using myoglobin (*m*/*z* 16,952.18).

### 3.4. Agglutination Studies

Agglutination was performed as described before, with minor alterations. Murine red blood cells for galectin-1 null mice were washed in phosphate-buffered saline, and a concentration range of oxidized human Gal-1, obtained at various incubation times with buffer containing 0.5 μM CuSO_4_, was tested. Data were quantified via light scattering at 650 nm using a plate reader.

## 4. Conclusions and Perspectives

HSQC-based monitoring has provided new structural evidence into the Gal-1 oxidation process, enabling us to refine a model for Gal-1 disulfide bond formation by integrating all available information on this process. Based on the structural proximity between cysteine residues and an oxidation-mediated increase in intramolecular dynamics, we propose that two disulfide bonds (C16-C88 and C42-C60) are formed during an initial, relatively slow oxidation phase. The predicted loss of four protons during this phase has been confirmed, and the dimer state and lactose affinity are maintained. Increases in motional dynamics appear to be a prerequisite for bringing the distant C2 and C130 residues together at the last stage of oxidation. Evidence for dynamic conformational interconversions likely explains why crystallization of Gal-1 following disulfide bridge formation has failed. When the C2 residue is unavailable, as in the Gal-1 C2S mutant, oxidation is arrested, and the nature of the C2/C130 configuration results from the relatively slow initial phase of the oxidation process. The fully oxidized Gal-1 dimer dissociates, lactose-binding capacity is lost, and a generally unfolded structure results. These events are confirmed by hemagglutination assays and monitoring Gal-1 properties via PFG NMR spectroscopy, results that are fully consistent with the previous literature on Gal-1 oxidation. The presence of DTT is effective at reverting oxidized Gal-1 into the reduced, active form of the lectin. Thus, Gal-1 contains a redox-dependent switch to its lectin activity. Interestingly, this process is the missing link for a context-dependent turn-off mechanism for Gal-1. What disulfides do for proto-type Gal-1 is accomplished irreversibly via proteolysis of chimera-type (Gal-3) and hetero-bivalent (Gal-8 and -9) galectins. The equivalents of the redox reaction are proteolytic truncation of Gal-3 to its CRD [52,53,54,55,56] and linker cleavage of tandem-repeat-type galectins that abolish their hetero-bivalency [57].

In physiological terms, it has been assumed that “if galaptin [a former term for galectin] has an extracellular function, then the lability of its binding activity in the presence of oxygen could place stringent restrictions on the latitude of its activity” [13]. Traveling along the classical secretory route within its oxidative environment has been revealed to fail to go beyond the endoplasmic reticulum when human Gal-1 was fused to signal peptide in human cells (HEK293), with Gal-1 co-fractionating and co-localizing with calnexin [58]. Thus, for the time, the cysteine-based switch has been preserved following non-classical secretion. When in an oxidative extracellular environment (such as sites of inflammation), the Gal-1 Cys thiols become sensors that “may be important in dictating the distribution and longevity of Gal-1 signaling” [59]. As these authors suggest, certain anti-inflammatory activities exerted by Gal-1 can be temporarily turned off by this oxidation mechanism, “allowing leukocytes to successfully neutralize pathogen or remove necrotic tissue”. The redox-driven process, thus, fine-tunes Gal-1 activity to the local needs, adding a chemical mode to the network of regulatory mechanisms for Gal-1 that also involves shifts in counter-receptor availability [60,61,62]. The realization of this concept provides good reason to systematically elucidate the activity of oxidized Gal-1 on leukocyte populations, considering details of the chemistry of the microenvironment. Equally important is that the local Gal-1 concentration also plays a pivotal role. In this context, the possibility of covalent association between two Gal-1 CRDs via inter-molecular C130-C130′ bonding (C130 being “among the residues that emerged during mammalian evolution” [63]), is also likely to affect the overall activity of oxidized Gal-1. In fact, covalent dimer engineering has been shown to increase the growth-inhibitory and anti-inflammatory activities of wild-type Gal-1 [64,65,66]. Examining what happens in detail under oxidative conditions in situ and to study the functionality of the oxidized Gal-1 states is a major challenge for future investigations.

Crystal structures of Gal-1 draw attention to a further structural aspect of oxidation, i.e., generation of sulfenate/sulfinate from thiol groups. In human Gal-1, C16 is oxidized to sulfenate in the wild-type protein and the C2S mutant, and in bovine Gal-1, sulfenate is seen at C130 and sulfinate at C16/C88 [39,67]. Since reactive oxygen species can also produce sulfonates, as detected at C106 in HMGB1 associated with the induction of tolerogenic properties [68], it is of interest to explore the fate of sulfhydryls in Gal-1 towards fully understanding their assumed roles as sensors, as well as the structural and functional diversity of oxidized Gal-1. Intra- and inter-molecular disulfide bond formation, as well as oxidization to sulfenate, sulfinate, and sulfonate, may cause functional consequences, warranting detailed analysis.

Overall, our present results reveal a dynamic and reversible impact of redox conditions on Gal-1 via the status of its six sulfhydryls. Thus, it is clear that deliberately elucidating the activity spectrum of oxidized Gal-1 states (via disulfide bonding, covalent bridging between CRDs, and the generation of sulfenate, sulfinate, and/or sulfonate) deserves further attention. Engineering of mutant cell lines (e.g., by implementing expressions of the C16S mutant instead of the wild-type protein) can help dissect the apparent complexity of Gal-1 functionality. These insights may then have potential for developing rational translational applications in various disease states, such as chronic inflammation or neurodegeneration, using various oxidized Gal-1 forms or bioinspired variants.

## Figures and Tables

**Table 1:** Kinetic parameters are derived from a double exponential fit of the recovery rate.

	Rate 1	Population 1	Rate 2	Population 2
All species	80 × 10^−3^ min^−1^	84%	0.8 × 10^−3^ min^−1^	16%
Native species	88 × 10^−3^ min^−1^	83%	10.3 × 10^−3^ min^−1^	17%

## Data Availability

Not applicable.

## Supplementary Materials

The supporting information can be downloaded at: https://www.mdpi.com/article/10.3390/ijms25136956/s1.

## Abbreviations

CRD, carbohydrate recognition domain; CG, chicken galectin; CD, circular dichroism; DSC, differential scanning calorimetry; FPLC, fast protein liquid chromatography; Gal-1, human galectin-1; HSQC, heteronuclear single quantum coherence; ITC; isothermal calorimetry; MS, mass spectrometry; NMR, nuclear magnetic resonance; PFG, pulsed field gradient.
