# Supplementary material for: Cysteine Oxidation in Human Galectin-1 Occurs Sequentially via a Folded Intermediate to a Fully Oxidized Unfolded Form
Source: Int J Mol Sci. 2024 Jun 25;25(13):6956. doi: 10.3390/ijms25136956 (PMC11241627; doi:10.3390/ijms25136956)

Gal-1 C2S, 0.65 mM apo at pH 7.3, 900 MHz 1D proton spectrum (DSS scaled).
Temp-dependent, reversible chemical exchange (26.8, 30.0, 33.0, 36.2 and 40.4 °C).

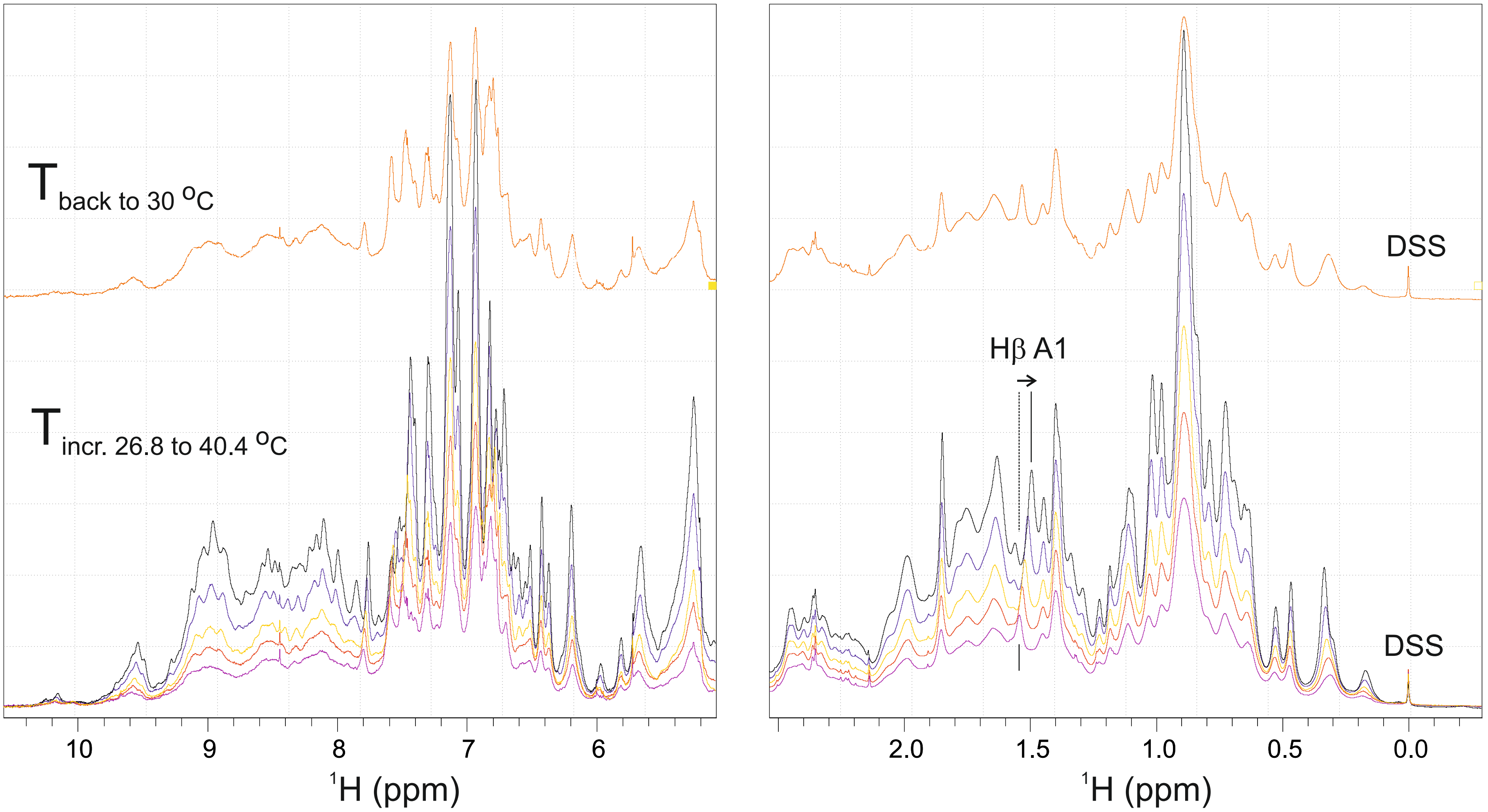

Supplement: Supplementary file 1 [file ijms-25-06956-s001.zip › Figure S6 Gal-1 C2S 22082012expno1-3-6-8-9 1D zgesgp tempseries.pdf]
