# Supplementary material for: Cysteine Oxidation in Human Galectin-1 Occurs Sequentially via a Folded Intermediate to a Fully Oxidized Unfolded Form
Source: Int J Mol Sci. 2024 Jun 25;25(13):6956. doi: 10.3390/ijms25136956 (PMC11241627; doi:10.3390/ijms25136956)
Supplement: Supplementary file 1 [file ijms-25-06956-s001.zip › ijms-3001763- supplementary.pdf]

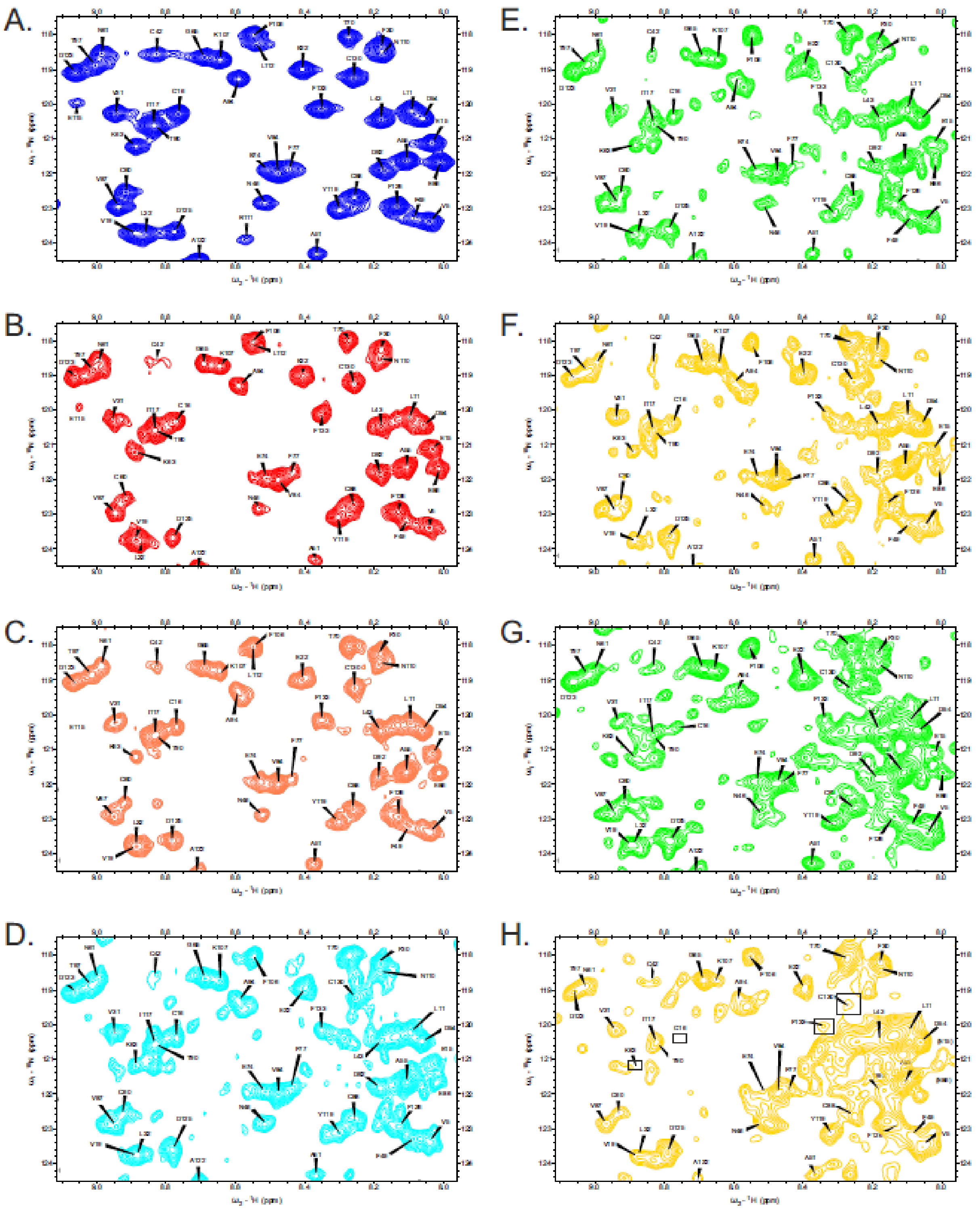

Fig. S1

Oxidation HSQC 15N-1H Gal-1 at 28 μM and 0.5 μM CuSO4, 30 oC, KPi pH 7.1, no DTT

Selected amide region containing all cysteine amides (except Cys2)

a. Reference Gal-1 28 uM in 8 mM DTT; b-g. oxidation series at timepoint b. 1.42 hr, c. 5.18 hr, d. 7.6 hr, e. 10.1 hr, f. 12.9 hr, g. 16.1 hr, and h. 23.5 hr.

Boxed peaks show intensity loss of amide cross peaks.

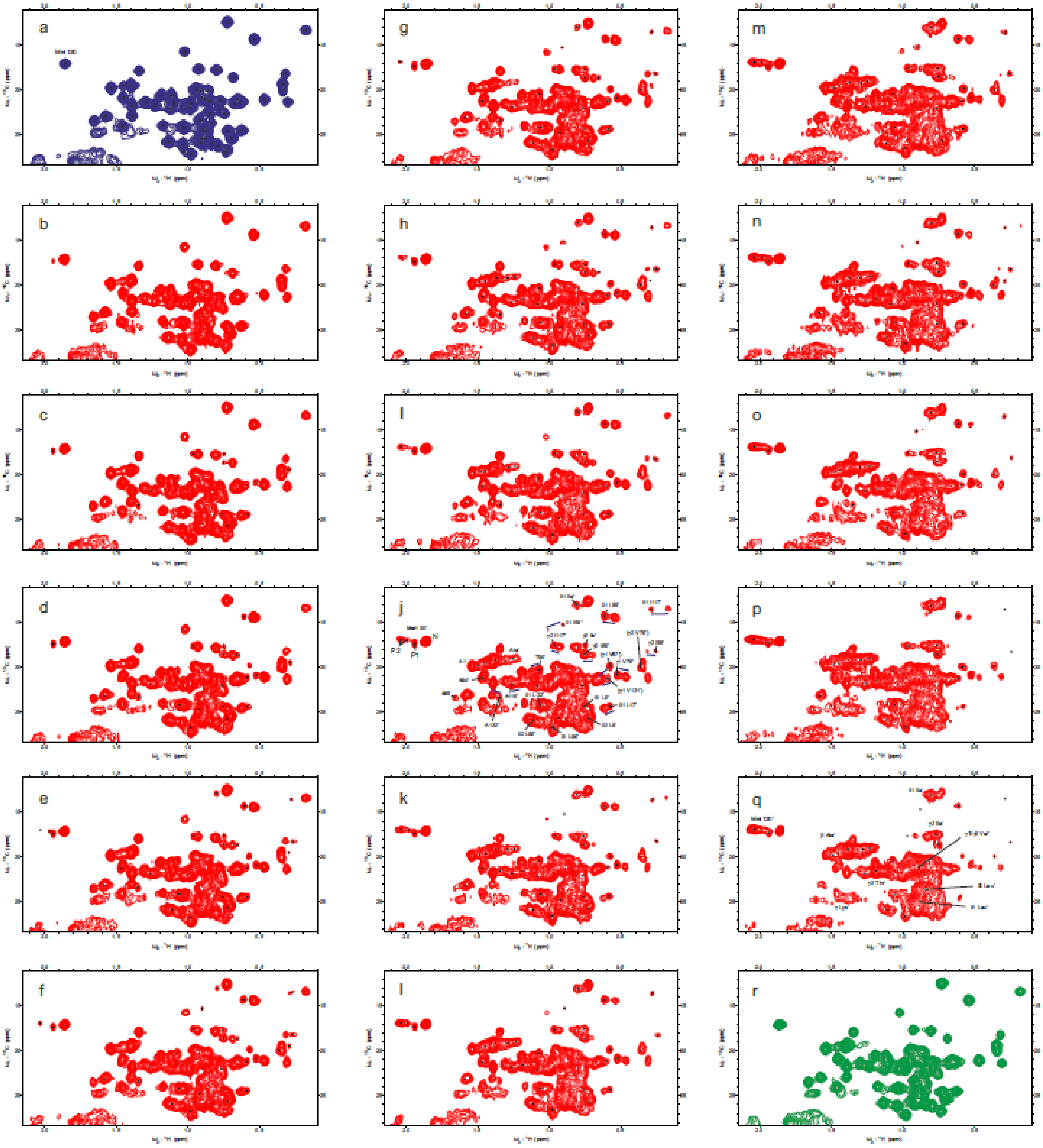

Fig. S2

A series of the part of 1H–13C HSQC spectra showing the methyl region of Gal-1 at different time points during oxidation with 0.5 µM CuSO4. a. Reference 30 uM Gal-1 [13C,15N] in 8 mM DTT; b-q. Gal-1

oxidation series at 0.5 μM CuSO4 , Time points range from resp. b. +1.47, c. +3.5, d. +5.6, e. +7.6, f. +9.7, g. +11.8, h. +13.8, i. +15.9, j.+17.9, k. +20.1, l. +23.7, m. +26.8, n. +29.9, o. +33.5, p +37.5 and q. +41.6 h. Following removal of CuSO4 and a 31 hr-long incubation in buffer containing 8 mM DTT and 2.5 μM EDTA, the next spectrum was measured ®. Some cross peaks are labeled. The concentration of $[^{13}C,^{15}N]$-labeled Gal-1 was 30 μM in 25 mM potassium phosphate buffer (pH 7.3), containing 0.5 μM CuSO4, the solution was incubated at 30 ^{o}C.

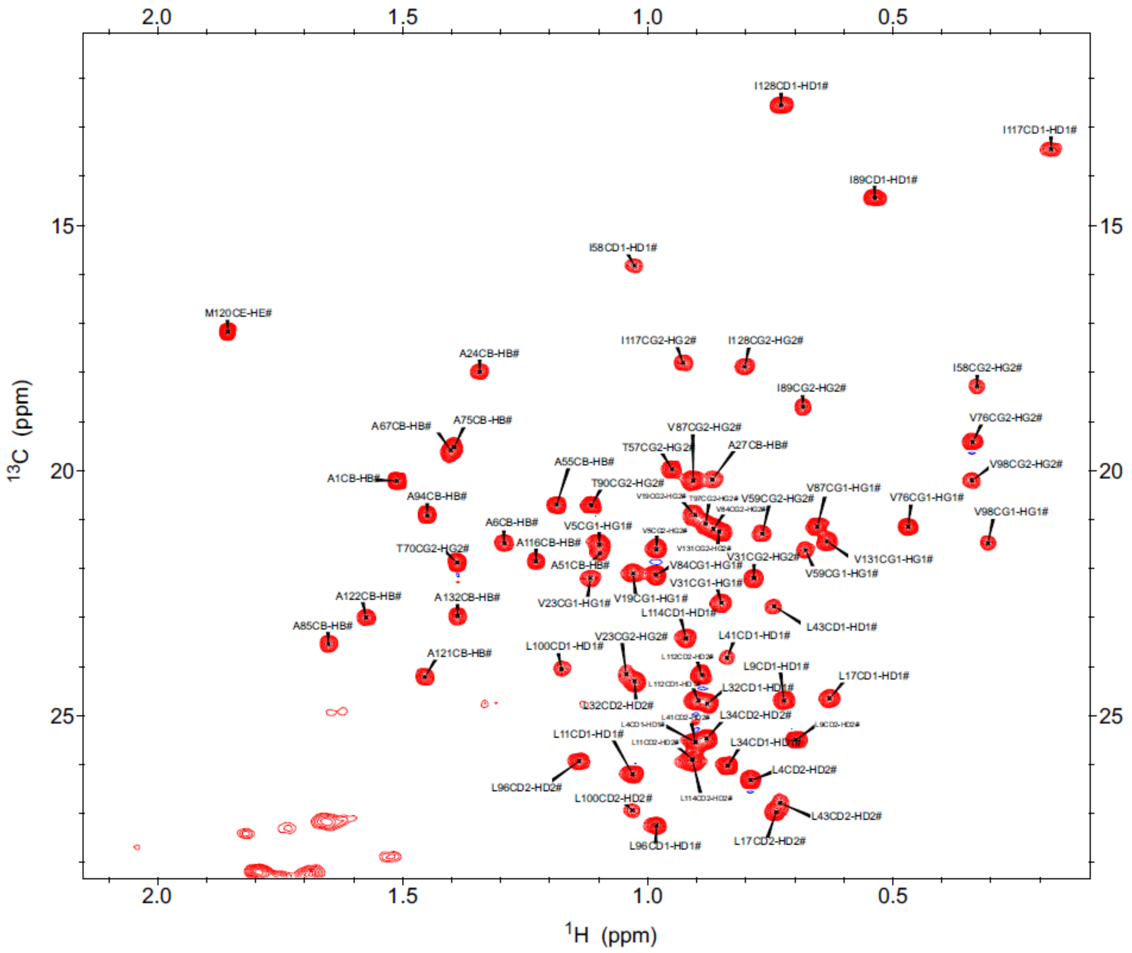

Fig. S3

The methyl region from a natural abundance 1H–13C HSQC spectrum of native 1 mM Gal-1 in 25 mM KPi buffer (pH 7.5) at 30 ^{o}C is shown. Labeled cross peaks have been assigned as indicated.

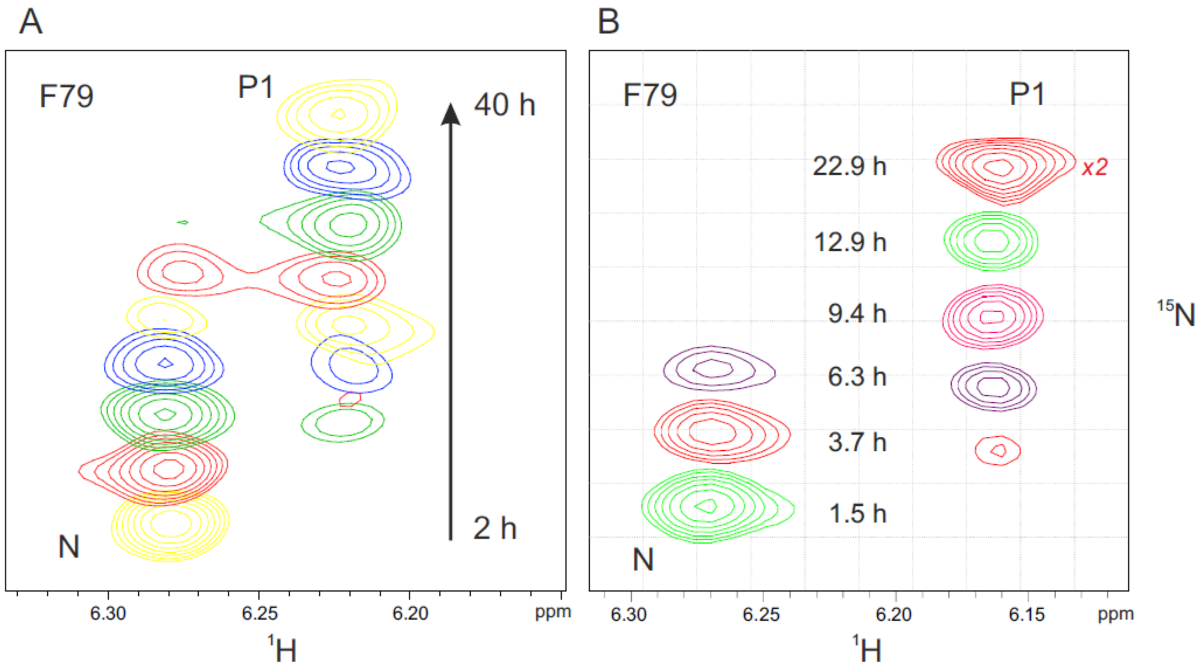

Fig. S4

a.The oxidation time course for Gal-1 C2S mutant N and P1 states from 2 to 40 hr. The 15N axis is shown as relative, because 1H–15N HSQC spectral expansions have been staggered in the 15N dimension in order to show time dependent changes in the intensities of N and P1 peaks in one plot. Signal intensities gradually drop during incubation of C2S, 2D contour levels have been lowered at higher incubation times to compensate for the loss.

b.Similar oxidation time course of Gal C16S (28 μM oxidation series at 4 μM CuSO4, 30 deg, 25 mM KPi pH 7.32). Under these conditions the C16S mutant give a more stable P1 state, so contour levels are set at fixed values here, except for the last time point where the minimum contour level is decreased by a factor of 2x at the point where all proton signal start to go down.

Extra:

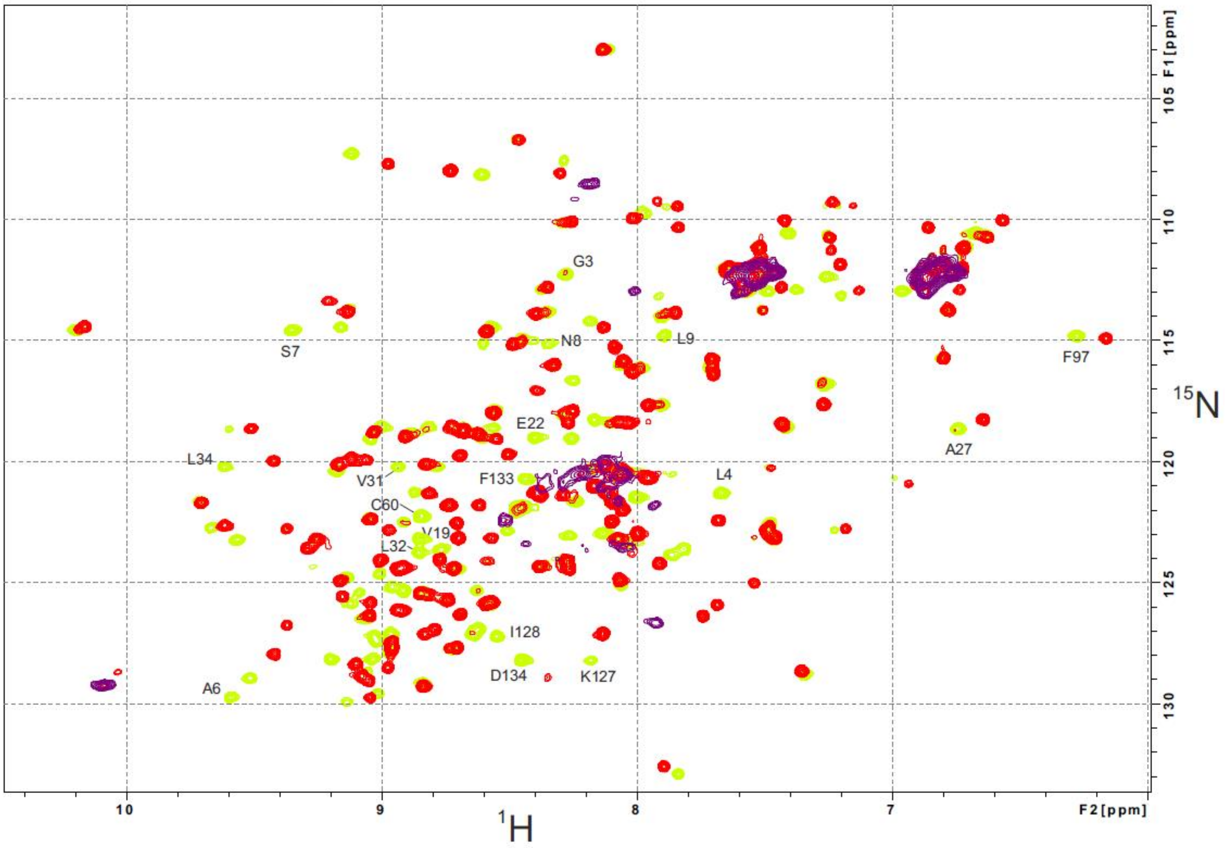

Fig. S5

2D HSQC 15N-1H spectrum of the metastable P1 state of oxidized [^{15}N]-Gal-1 C16S mutant protein (28 μM Gal-1 C16S, at 30 oC in 25 mM KPI buffer, pH 7.32, plus 4 μM CuSO4). Three plotted HSQC spectra are overlayed; the initial fresh solution before addition of CuSO4 (yellow), the spectrum after 11 h incubation time in 4 μM CuSO4 that gives a nearly conformational pure P1 state (red), and the broadened out final spectrum after incubation, recorded after ca. 33 h (purple).

Gal-1 C2S, 0.65 mM apo at pH 7.3, 900 MHz 1D proton spectrum (DSS scaled).
Temp-dependent, reversible chemical exchange (26.8, 30.0, 33.0, 36.2 and 40.4 °C).

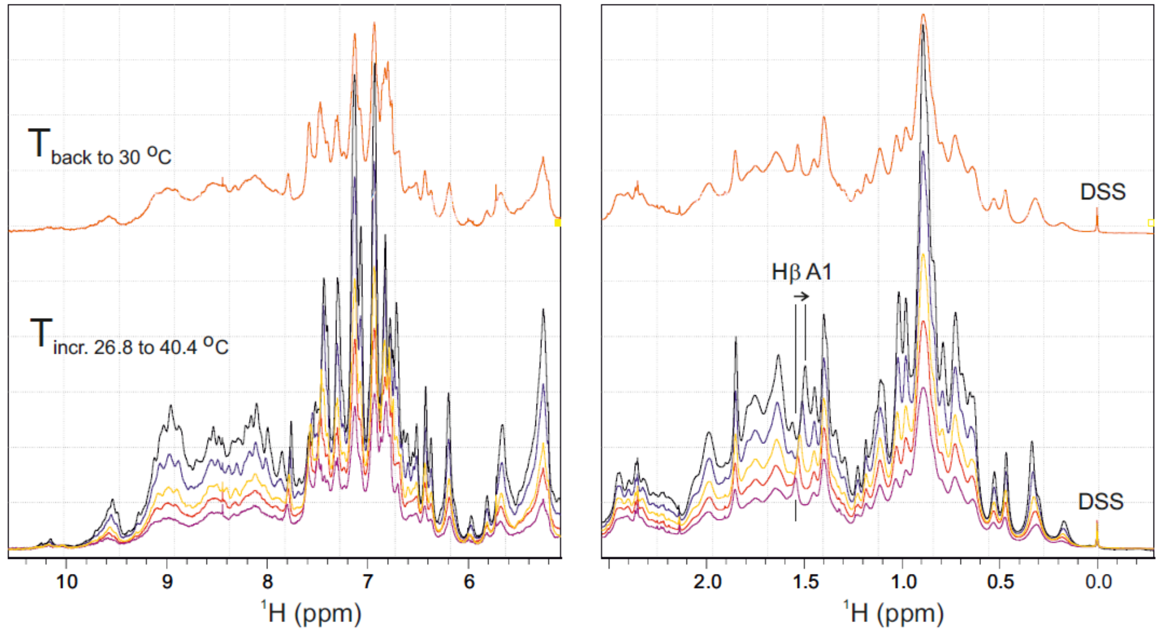

Fig. S6.

Strong temperature dependency of 1D 1H spectra for Gal-1 C2S, reversible conformational behavior.
