# Supplementary figures and images for: Cysteine Oxidation in Human Galectin-1 Occurs Sequentially via a Folded Intermediate to a Fully Oxidized Unfolded Form
Source: Int J Mol Sci. 2024 Jun 25;25(13):6956. doi: 10.3390/ijms25136956 (PMC11241627; doi:10.3390/ijms25136956)

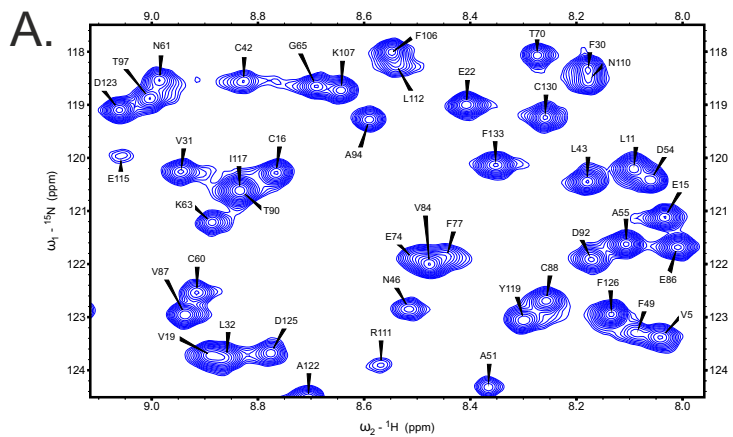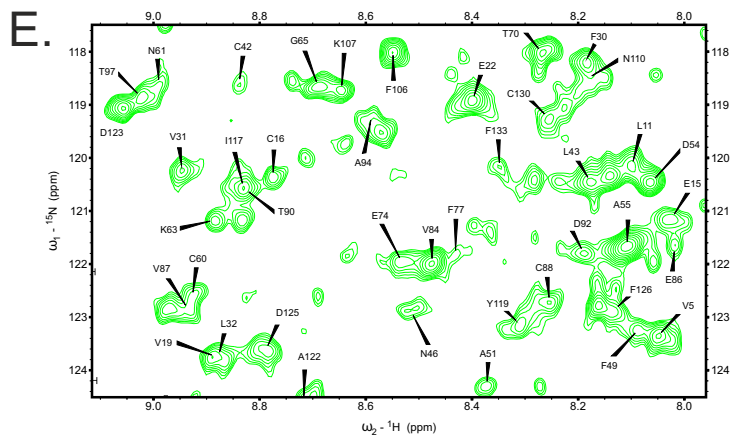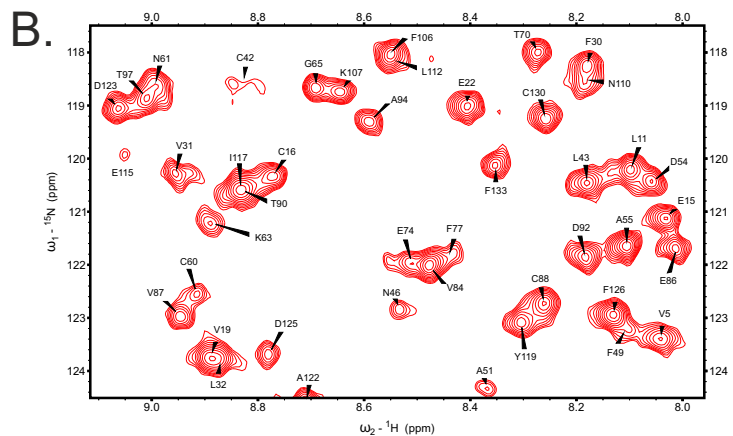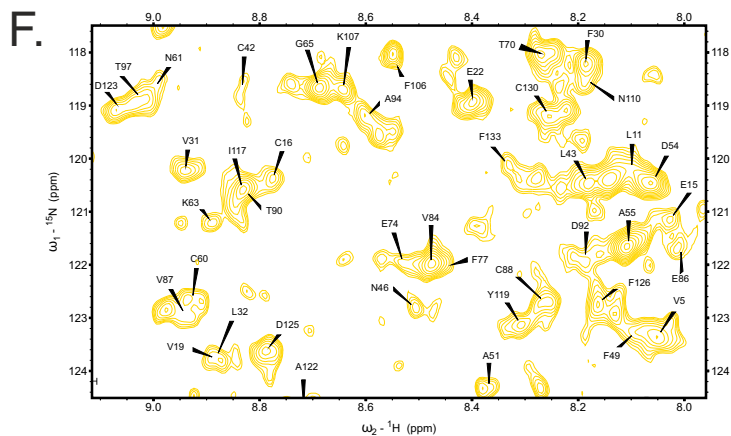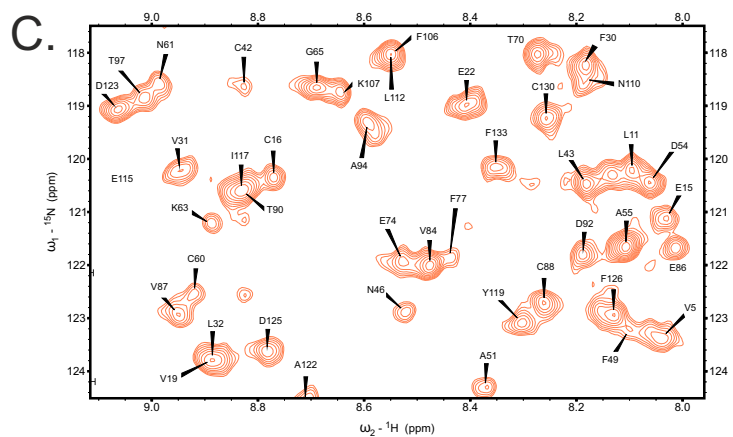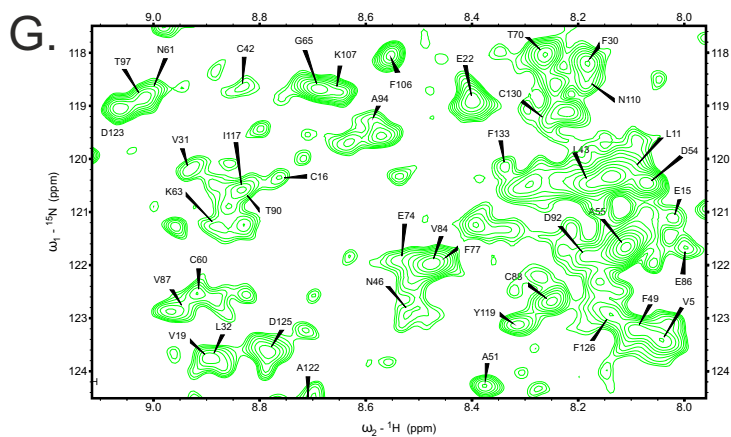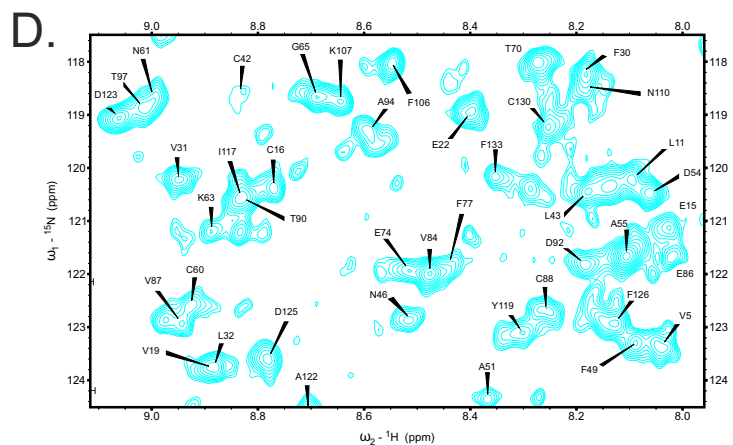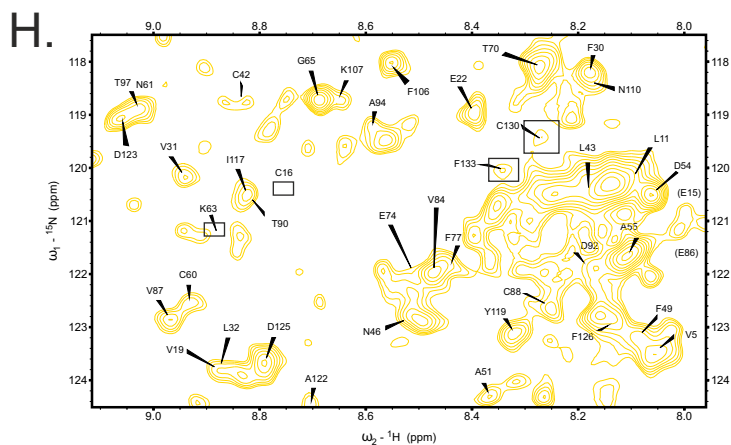

Supplement: Supplementary file 1 [file ijms-25-06956-s001.zip › Figure S1 HSQC 15N-1H oxidation series.pdf]

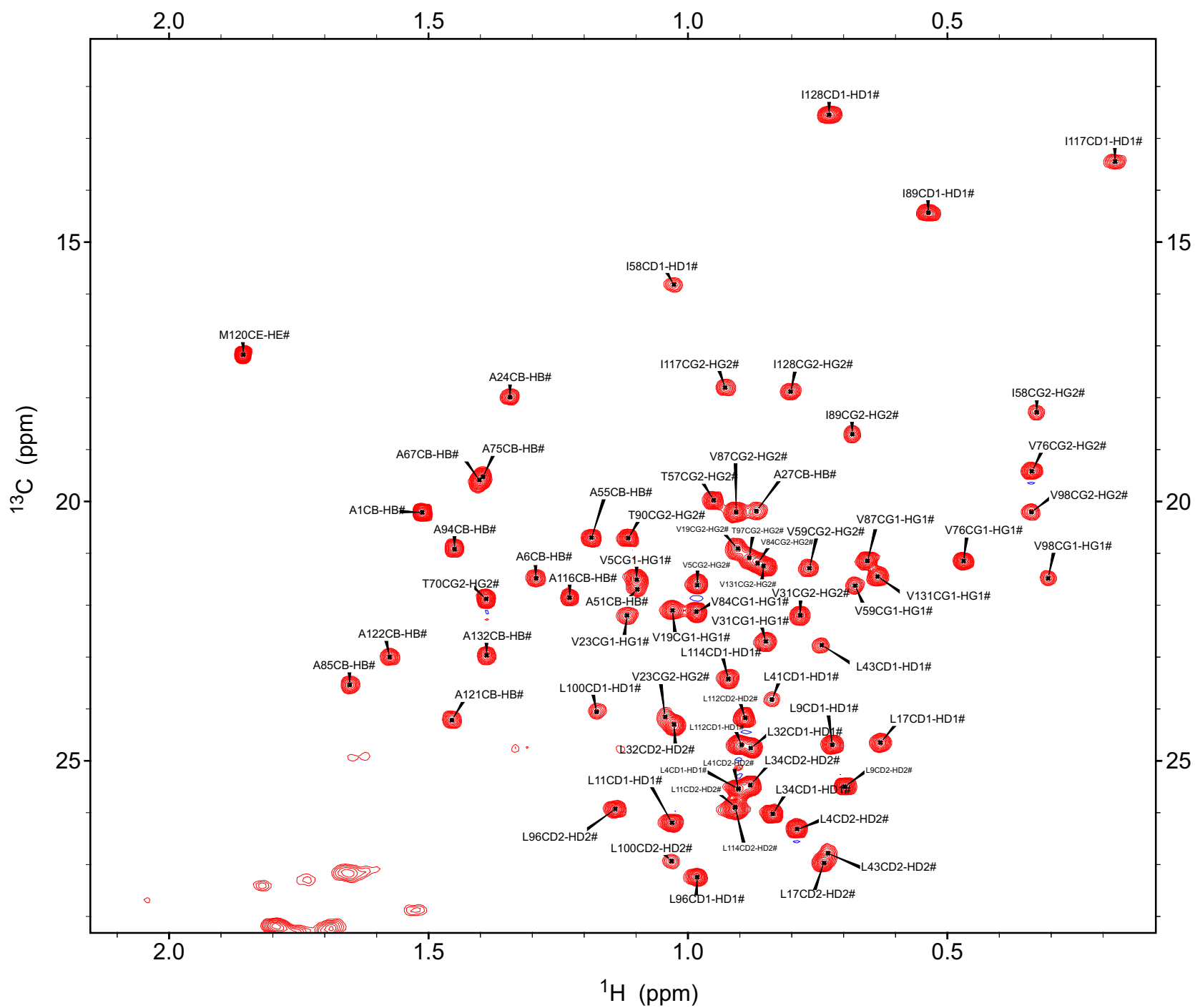

Supplement: Supplementary file 1 [file ijms-25-06956-s001.zip › Figure S3 Gal-1 at 1 mM conc nat abund HSQC 13C-1H NMR assignment.pdf]

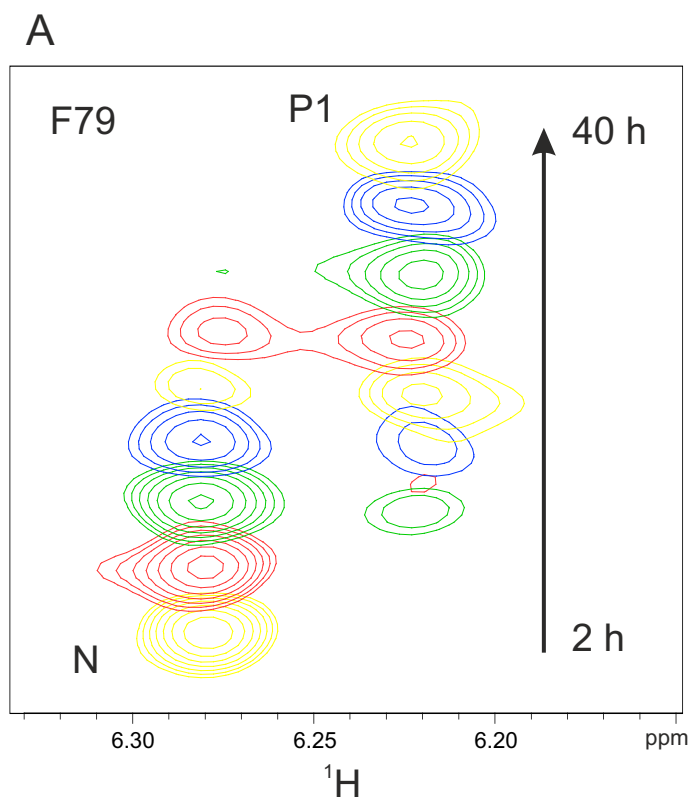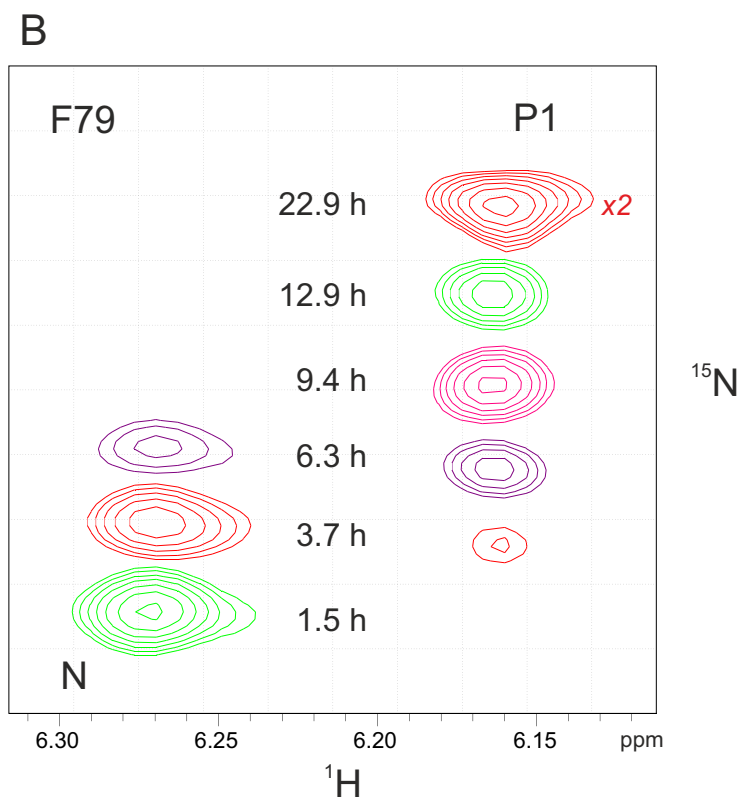

Supplement: Supplementary file 1 [file ijms-25-06956-s001.zip › Figure S4 LSF_HI_Gal1 C2S and C16S HSQC15N F79 timeseries2D.pdf]

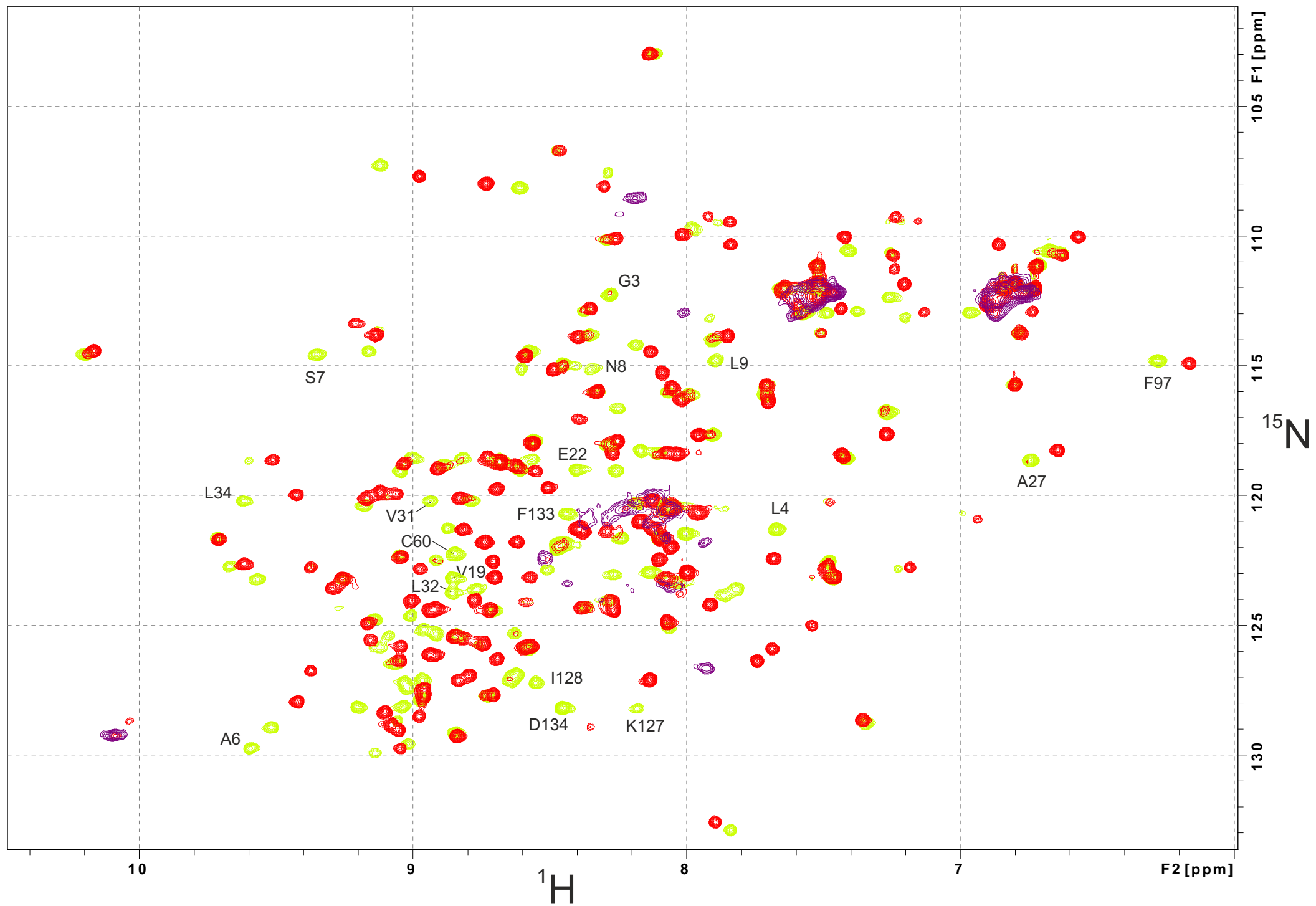

Supplement: Supplementary file 1 [file ijms-25-06956-s001.zip › Figure S5 LSF_HI_GalC16S_17092012expno16-5-23_differenceHSQCnative and P1.pdf]
