# Supplementary material for: Cysteine Oxidation in Human Galectin-1 Occurs Sequentially via a Folded Intermediate to a Fully Oxidized Unfolded Form
Source: Int J Mol Sci. 2024 Jun 25;25(13):6956. doi: 10.3390/ijms25136956 (PMC11241627; doi:10.3390/ijms25136956)
Supplement: Supplementary file 1 [file Figure S2 Gal-1 [13C15N] HSQC 13C-1H NMR oxidation.pdf › Figure S2 Gal-1 [13C15N] HSQC 13C-1H NMR oxidation.pdf]

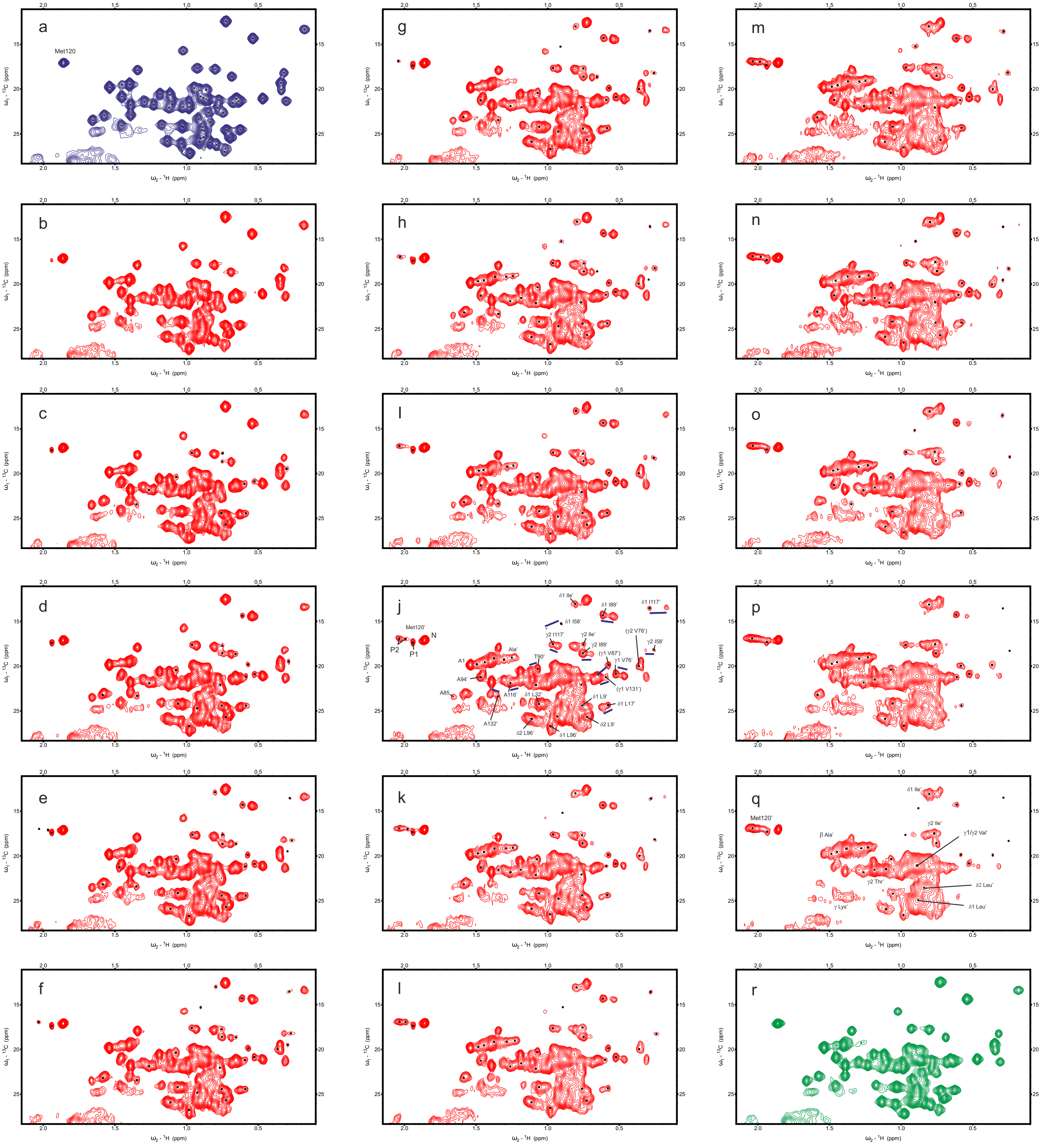
a
Met120
b
c
d
e
f
g
h
i
j
Met120'
N
P2
P1
A1
Ala'
A94'
A85
A116'
A132'
T90'
δ1 Ile'
δ1 I89'
δ1 I117'
δ1 I58'
γ2 I117'
γ2 Ile'
γ2 I89'
(γ2 V76')
γ2 I58'
(γ1 V87')
γ1 V76'
(γ1 V131')
δ1 L32'
δ1 L9'
δ1 L17'
δ2 L9'
δ2 L96'
δ1 L96'
k
l
m
n
o
p
q
δ1 Ile'
Met120'
γ2 Ile'
β Ala'
γ1/γ2 Val'
γ2 Thr'
δ2 Leu'
γ Lys'
δ1 Leu'
r
ω1 - 13C (ppm)
ω2 - 1H (ppm)
